# Differential effects of attention and contrast on transition appearance during binocular rivalry

**DOI:** 10.1167/jov.26.1.14

**Published:** 2026-01-23

**Authors:** Cemre Yilmaz, Kerstin Maitz, Maximilian Gerschütz, Wilfried Grassegger, Anja Ischebeck, Andreas Bartels, Natalia Zaretskaya

**Affiliations:** 1 Department of Psychology, University of Graz, Graz, Austria; 2 BioTechMed-Graz, Graz, Austria; 3 Department of Psychology, University of Tübingen, Tübingen, Germany

**Keywords:** binocular rivalry, contrast, attention, transition, visual perception

## Abstract

Binocular rivalry occurs when two eyes are presented with two conflicting stimuli. Although the physical stimulation stays the same, the conscious percept changes over time. This property makes it a unique paradigm in both vision science and consciousness research. Two key parameters, contrast and attention, were repeatedly shown to affect binocular rivalry dynamics in a similar manner. This was taken as evidence that attention acts by enhancing effective stimulus contrast. Brief transition periods between the two clear percepts have so far been much less investigated. In a previous study we demonstrated that transition periods can appear in different forms depending on the stimulus type and the observer. In the current study, we investigated how attention and contrast affect transition appearance. Observers viewed binocular rivalry and reported their perception of the four most common transition types by a button press while either the stimulus contrast or the locus of exogenous attention was manipulated. We show that contrast and attention similarly affect the overall binocular rivalry dynamics, but their effects on the appearance of transitions differ. These results suggest that the effect of attention is different from a simple enhancement of stimulus strength, which becomes evident only when different transition types are considered.

## Introduction

Binocular rivalry is a type of bistable visual stimulus where the two eyes see completely different, conflicting images. As a result, the conscious percept alternates between clear images, with brief transition phases between them. The alternation rate and duration of clear percepts are important parameters of binocular rivalry dynamics that are used to probe various aspects of visual processing, like stereopsis (Blake & Wilson, 2011), plasticity (Castaldi, Lunghi, & Morrone, 2020), and perceptual learning (Klink, Brascamp, Blake, & van Wezel, 2010). Changes in these parameters have also been associated with typical development and aging (Arani, van Ee, & van Wezel, 2019; Beers, Slugocki, Lewis, Sekuler, & Bennett, 2015; Hudak et al., 2011; Ukai, Ando, & Kuze, 2003), as well as with psychiatric (Miller et al., 2003; Nagamine, Yoshino, Miyazaki, Takahashi, & Nomura, 2009; Ye, Zhu, Zhou, He, & Wang, 2019) and neurodevelopmental (Amador-Campos, Aznar-Casanova, Ortiz-Guerra, Moreno-Sánchez, & Medina-Peña, 2015; Casanova, Campos, Sánchez, & Supèr, 2013; Dunn & Jones, 2020; Jusyte, Zaretskaya, Höhnle, Bartels, & Schönenberg, 2018; Karaminis, Lunghi, Neil, Burr, & Pellicano, 2017; Robertson, Kravitz, Freyberg, Baron-Cohen, & Baker, 2013) disorders. Furthermore, despite criticism (Blake, Brascamp, & Heeger, 2014), binocular rivalry remains one of the key paradigms in studying the neural mechanisms of conscious perception (Carmel, Walsh, Lavie, & Rees, 2010; de Jong et al., 2020; Gelbard-Sagiv, Mudrik, Hill, Koch, & Fried, 2018; Kapoor et al., 2022; Lumer, Friston, & Rees, 1998; Tong, Nakayama, Vaughan, & Kanwisher, 1998; Zaretskaya, Thielscher, Logothetis, & Bartels, 2010). In sum, binocular rivalry represents a key experimental paradigm in both vision science and consciousness research, with potential clinical relevance.

Two key factors, bottom-up stimulus strength and attention, have been frequently and reliably reported to influence binocular rivalry dynamics (Brascamp, Klink, & Levelt, 2015; Levelt, 1966; Paffen & Alais, 2011). Increasing the contrast or luminance of both stimuli (collectively termed “stimulus strength”) leads to a faster overall alternation rate (Naber et al., 2020; Paffen, Alais, & Verstraten, 2006). At the same time, increasing the strength of one of the stimuli primarily increases the dominance balance towards the stronger stimulus (Brascamp et al., 2015) (although recent studies also showed that these rules do not hold for rivalry between contrast- and luminance-modulated stimuli; Skerswetat, Formankiewicz, & Waugh, 2018a; Skerswetat, Formankiewicz, & Waugh, 2020). In parallel to stimulus strength, focusing attention on the rivaling stimuli speeds up binocular rivalry (Paffen & Van der Stigchel, 2010) while diverting attention away from the rivalry stimuli slows it down (Alais, van Boxtel, Parker, & van Ee, 2010; Paffen et al., 2006) or even leads to a complete stop of binocular rivalry alternations (Brascamp & Blake, 2012; Zhang, Jamison, Engel, He, & He, 2011). Accordingly, attention to one of the stimuli can change the dominance balance between the two competing percepts (Moreno-Sánchez, Aznar-Casanova, & Valle-Inclán, 2019; Ooi & He, 1999). This similarity between the effects of stimulus strength and attention led researchers to conclude that attention acts by increasing the stimulus strength, e.g., effective contrast, of the rivalry stimuli (Paffen & Alais, 2011). This fits well with a broader notion of the effects of attention in the visual system, which is thought to enhance the perceived contrast of the stimuli (Carrasco, Ling, & Read, 2004; Carrasco & Barbot, 2019).

More recently, there has been increasing interest in transition periods between the two dominant percepts in binocular rivalry. This interest is motivated by the acknowledgement that transition periods are not instantaneous, and the times when a combination of both images is perceived can represent another important characteristic of the visual system dynamics (Brascamp, Van Ee, Noest, Jacobs, & Van Den Berg, 2006). For example, symptom severity in adult attention deficit hyperactivity disorder (ADHD) and autism spectrum disorder (ASD) has been specifically linked to an increase in the duration of transition periods (Casanova et al., 2013; Dunn & Jones, 2020; Karaminis et al., 2017; Robertson et al., 2013; Spiegel, Mentch, Haskins, & Robertson, 2019; but see Said, Egan, Minshew, Behrmann, & Heeger, 2013), more prominently for superimposed but not for piecemeal transitions (Skerswetat, Bex, & Baron-Cohen, 2022 for ASD), and has been interpreted as a marker of shallow inhibition in the visual system in these disorders (Robertson, Ratai, & Kanwisher, 2016). In contrast to dominance durations and reversal rate (the number of changes from one stimulus to the other in one second), only the effect of contrast on transition duration has been reported so far, with reduction of transition duration as a result of increasing stimulus contrast (Brascamp et al., 2006; Hollins, 1980), and an increase in superimposed transition with increasing visibility of contrast-modulated gratings (Skerswetat et al., 2018a).

Transitions during binocular rivalry are commonly assumed to be either a sudden and immediate switch between the two images or a brief period of perceiving a mixture of the two images. However, there are reports indicating that the actual experience during transition periods can vary. For example, “piecemeal” (Blake, O'Shea, & Mueller, 1992; Panum, 1858 as cited by Hollins, 1980), “superimposed” (Ogle & Wakefield, 1967 as cited by Hollins, 1980; Yang, Rose, & Blake, 1992), and “traveling wave” (Wilson, Blake, & Lee, 2001) transitions have been reported. In addition, the summation of two stimuli at the beginning of rivalry was suggested as a separate form of mixed perception other than these ‘false fusion’ transitions (Liu, Tyler, & Schor, 1992).

In previous studies, piecemeal and superimposed transitions were already included in the experimental paradigm with retrospective reports (Klink et al., 2010), separate buttons on a response box (Skerswetat, Formankiewicz, & Waugh, 2018b; Sheynin, Proulx, & Hess, 2019) or a joystick, which allowed to find an indication that some transition types may be more affected by contrast (Skerswetat & Bex, 2023). In our recent study, we systematically examined the subjective experience of transitions during binocular rivalry in healthy adult volunteers by allowing them to describe their perception verbally and with simple sketches, and showed that transitions appear in different forms depending on the observer and the exact stimuli used to induce binocular rivalry (Yilmaz et al., 2025). The most frequent transition types reported by at least half of participants in this study were superimposed (superimposition of the two images), piecemeal (parts of the two images distributed across the stimulus extent), traveling waves (one image uncovers the previously suppressed image like a “curtain”) and immediate transitions (instantaneous switch between the two images). The fact that transitions appear in different forms raises the question of whether the two key factors, contrast and attention, that similarly influence overall binocular rivalry dynamics, also similarly affect different transition appearances.

In the current study, we performed two experiments to investigate the effect of contrast and attention on transition appearance. In our design, we flipped the classical binocular rivalry task: observers pressed one of the four buttons to report which type of transition they were perceiving during binocular rivalry and released all buttons during the dominant percept or any other perceptual state. As in our previous study (Yilmaz et al., 2025), we define a transition as any perceptual state other than the exclusive perception of one of the images. This includes all types of intermediate percepts that represent a mixture of two images as well as the immediate changes from one to the other dominant percept. Therefore the term “transition” in our study does not necessarily imply a perceptual switch between two dominant percepts. In the first experiment, we manipulated the contrast of either one or both stimuli to test its effect on the relative frequency (fraction) and duration of the four main transition types. In the second experiment, we directed spatial attention either to one of the rivalry stimuli, to both, or to the fusion frame surrounding the stimuli.

## Experiment 1: Contrast

### Methods and material

The main hypotheses, experimental procedures and data analysis strategy were preregistered before the start of the data collection on osf.io (https://osf.io/6tvep).

#### Observers

A total of 42 observers were recruited for the study. After applying the exclusion criteria (see “Screening and exclusion criteria” section below), data from a total of 36 observers between 19 and 38 years old (*M* = 24, *SD* = 4.5, 27 female, eight male, one diverse) were used in the final analysis. We ensured that the sample size was sufficient with an a priori power analysis for our primary hypothesis (the effect of contrast condition on transition appearance) using G*Power (Faul, Erdfelder, Lang, & Buchner, 2007; Faul, Erdfelder, Buchner, & Lang, 2009). The following settings were used: repeated measures analysis of variance (ANOVA) for one within-subject factor with 3 levels, 80% power, assumed effect size of 0.25, assumed correlation of 0.5 among repeated measures, false positive probability of 0.05, assuming sphericity. The power analysis yielded a required sample size of 28 observers to reject the null hypothesis. All the observers gave their written informed consent and agreed with the data protection policy prior to participating. The study was positively assessed by the ethics committee of the University of Graz.

#### Screening and exclusion criteria

Before the main experiment, observers underwent a screening procedure to ensure they fulfilled the inclusion criteria. All major exclusion criteria were set before data collection as part of the study preregistration. With the best correction (wearing contact lenses or eye glass if prescribed), they first performed the Freiburg Visual Acuity Test $FrACT_{10}^{2022-04-26}$ (https://michaelbach.de/ot/FrACT10/capp/index.html; Bach, 1996) with both eyes open and then a Random Dot V Stereo Test (http://www.neuro-o.se/CritVis/cVis2.html#3DV). Both tests were presented on a VIEWPixx 3D monitor (1920 px × 1200 px, diagonal display size: 22.5 inches, vertical refresh rate: 120 Hz, viewing distance: 125 cm; VPixx Technologies, Saint-Bruno-de-Montarville, QC, Canada) used in monocular mode.

Observers were excluded if they had low visual acuity (logMAR > 0.4), poor stereovision (stereoacuity < 30 arcsec) or strong eye dominance (>0.8 dominance of one eye in each block). Eye dominance was used as an exclusion criterion to avoid floor effects in the number of transitions. Observers with strong eye dominance would perceive few (if any) transitions, and the higher contrast level of stimuli or attention to the stronger eye would reduce the transition number even further. Eye dominance was determined based on binocular rivalry reporting in the training session (see sections “Experimental design” and “Calculation of eye dominance” below). Furthermore, the accuracy of observers in their report of conscious perception during a replay block was used as a measure of response reliability (see “Binocular rivalry and replay analysis” for details). Data of observers that show up as outliers in replay accuracy score were considered unreliable. We analyzed the accuracy scores for potential outliers by applying the 1.5 interquartile range (IQR) rule (Hyndman & Fan, 1996; Tukey, 1977), resulting in the exclusion of one of the observers. In addition to these exclusion criteria, two observers experienced double vision (i.e., two stimulus apertures could not be fused resulting in a perception of two stimuli side by side). In total, six observers were excluded based on the criteria described above (double vision: 2, visual acuity: 3, response accuracy: 1).

### Experimental design

The experiment consisted of one training and three experimental sessions. In the training session, observers underwent the prescreening tests (see “Screening and exclusion criteria”), followed by three practice blocks with a classical binocular rivalry task (reporting percepts A and B by pressing and holding one of the two corresponding buttons), one block for each stimulus pair. After the practice blocks, they completed the same task again for each of the three stimulus pairs for the calculation of eye dominance.

#### Procedure and experimental conditions

**Figure 1.**
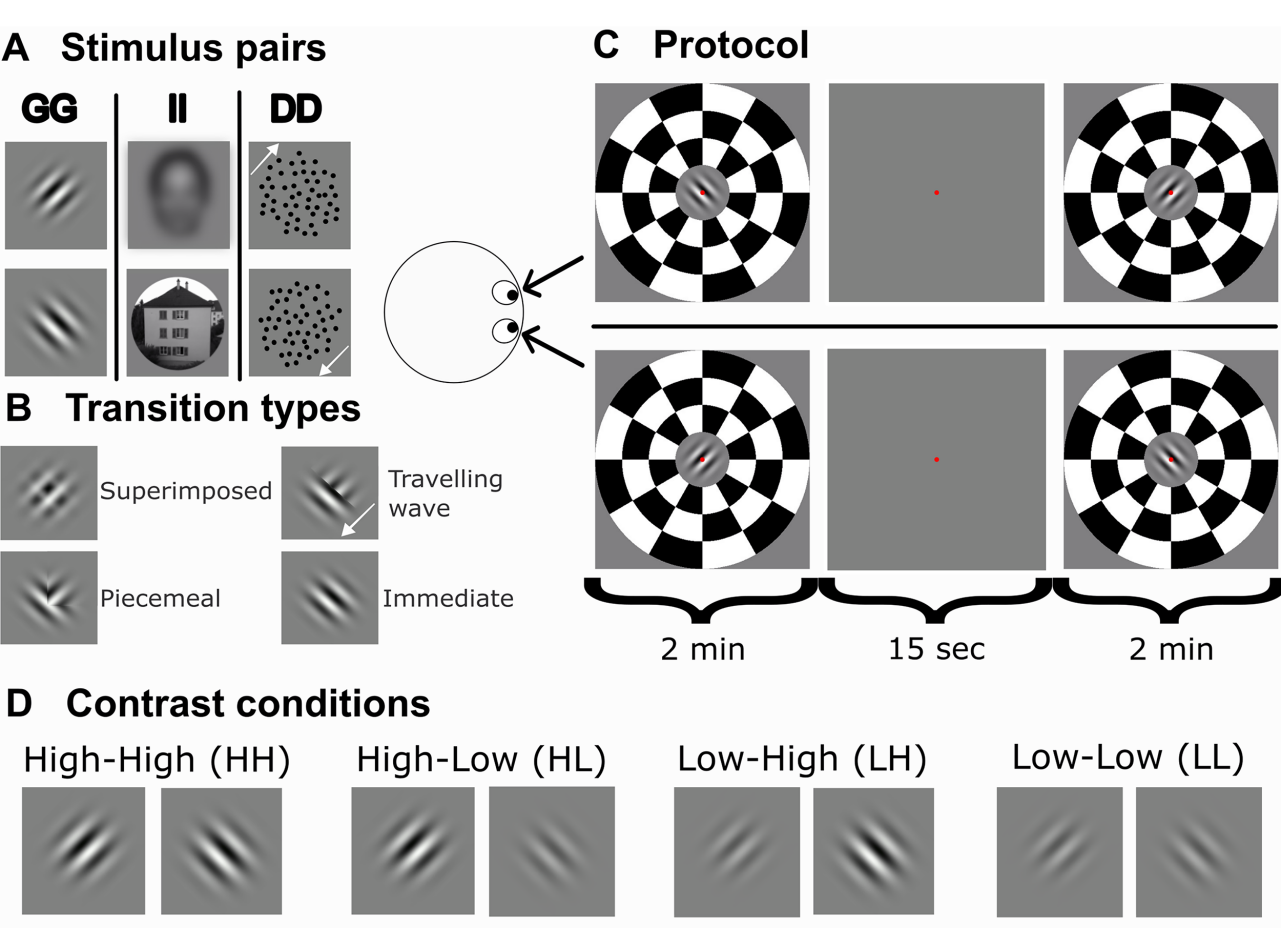
**Experimental protocol.** The observers participated in three experimental sessions, during which they were presented with different stimulus pairs at different contrast levels. (**A**) In each session, one of the three stimulus pairs was presented: gratings (GG), images (II), or moving dots (DD). The image of face blurred for anonymity. (**B**) During the task, the observers pressed a button to report which of the four most frequent transition types they were perceiving: superimposed, piecemeal, traveling wave, and immediate. (**C**) In one block, the eye-stimulus combination was counterbalanced between two 2-minute trials with a 15-second break. (**D**) Each stimulus pair was presented at four different contrast conditions: high-high (HH), high-low (HL), low-high (LH), and low-low (LL).

In each of the three experimental sessions, observers were presented with one of the three different stimulus pairs in a pseudorandomized order: (1) orthogonal sinusoidal gratings, (2) images of a face and a house, and (3) dot clouds moving in opposite directions (Figure 1A). Within each session, the same stimulus pair was presented at four different contrast levels: both stimuli at high contrast (HH), stimulus A at high and stimulus B at low contrast (HL), stimulus A at low and stimulus B at high contrast (LH), both stimuli at low contrast (LL) as illustrated in Figure 1D.

Stimuli were presented in blocks, each containing two 2-min trials with a 15-second break. The two trials counterbalanced the eye-stimulus combination (see Figure 1C). Each session consisted of 11 blocks: one training block that was subsequently discarded, two repetitions of four contrast pairs in a pseudorandomized order (2 × 4 blocks), and two replay blocks. In the replay blocks, the observers were presented with an imitation of rivalry derived from the data of a pilot observer, which simulated abrupt and smooth superimposed transitions (see Jusyte et al., 2018 for details). Only superimposed and immediate transitions (defined as short superimposed transitions) were included in the replay as this sufficed for assessing response accuracy and the ecological validity of mimicked transitions is limited. Piecemeal, traveling waves (and other transition variations) were not presented. The replay blocks included pairs of high-high and low-low contrast levels. Reports of observers during replay were subsequently used to quantify the observers’ accuracy in reporting perceptual changes.

#### Task

Observers were asked to report superimposed (S), piecemeal (P), traveling wave (T) and immediate (I) transitions (Figure 1B), which were the most frequent transition types experienced by observers in our previous study (Yilmaz et al., 2025). During stimulus presentation, they pressed one of the four buttons to report the transition for as long as the transition type lasted on a Cedrus RB-730 response box (seven tactile keys, USB interface, <1 ms temporal precision; Cedrus Corporation, San Pedro, CA, USA) and released the button during the onset of a clear percept or any other transition type. In contrast to the classical binocular rivalry task, the observers didn't press any button during dominant percepts.

Although this approach to reporting binocular rivalry hinders the measurement of dominance durations, we deliberately chose it for our current research question, which focuses on transitions. Although our response device with seven buttons allows us to collect seven different responses, observers at the piloting stage had difficulty selecting from more than four responses options. Another promising approach has been recently developed to report perceptual experience during binocular rivalry in a non-binary fashion using a joystick (Skerswetat & Bex, 2023). However, this approach allows us to measure the properties of two different transition types simultaneously with exclusive percepts. In the current study, we wanted to capture the parameters of as many transition types as possible. Therefore we deliberately chose a discrete four-button approach, with each button corresponding to one of the most frequent transition types.

We would also like to note that the instruction of reporting only four different transition types is likely to yield much lower estimates of the transition rate than what is typically observed in classical binocular rivalry studies (e.g., Alais et al., 2010; Levelt, 1965; Paffen et al., 2006). In a typical binocular rivalry study, the subjects are asked to report exclusive percepts and not to press any button when unsure or during a mixed percept. Hence, a typical binocular rivalry study is designed to accurately measure periods of exclusivity but is in a way biased to yielding more frequent “transition” periods when subjects are unsure. In this study, observers reported only the four specific types of transitions, and did not press any button for exclusive percepts, all other transition types, and uncertainty periods. Therefore, although our paradigm is tailored at accurate reporting the four transition types of interest, it might underestimate the actual transition rate.

#### Stimuli

All stimuli for the main experiment were generated using MATLAB 2024b (MathWorks, Inc., Natick, MA, USA) and Psychtoolbox (Brainard, 1997; Kleiner, Brainard, & Pelli, 2007; Pelli, 1997) on a Linux Ubuntu 22.04 LTS computer, and presented on a linearized VIEWPixx 3D monitor (1920 px × 1200 px, diagonal display size: 22.5 inches, vertical refresh rate: 120 Hz, viewing distance: 80 cm; VPixx Technologies). Dichoptic stimulation was achieved using a shutter glasses system (3DPixx LCD shutter glasses, VPixx Technologies), which includes two pairs of 3D EDGETM VR glasses and an ActiveHubRF50TM RF emitter. The system was synchronized with the monitor's vertical refresh rate by the RF emitter, yielding an effective refresh rate of 60 Hz per eye.

Throughout all experiments and conditions, stimuli were presented foveally and were surrounded by a checkerboard ring, which was identical for the two eyes to aid stable vergence. The checkerboard ring extended between 3.8° and 15.2° of eccentricity. A red fixation dot at the center (0.18° in diameter) was also displayed superimposed on both stimuli. Each stimulus was 3.8° in diameter.

We used three stimulus types commonly used in binocular rivalry studies (Figure 1A): (1) orthogonal static sinusoidal gratings tilted ±45° from the vertical orientation with a spatial frequency of 1.26 c/° within a Gaussian envelope, (2) images of a face and a house, and (3) dots moving diagonally (i.e., either left-down or right-up) with a speed of 0.40°/sec. Each frame had a dot density of 13 dots/°, and each dot had a 0.12° diameter. To prevent observers from tracking individual dots, we restricted the lifetime of each dot to 60 frames per eye. For gratings, the contrast was set to 90% Michelson contrast, for images to 0.4 RMS contrast, and the contrast for the dots was set to 0.90 Weber contrast for the high contrast level. For the low contrast level, the contrast was set to 30% Michelson contrast for gratings, for images to 0.1 RMS contrast, and the contrast for the dots was set to 0.30 Weber contrast.

### Data analysis

All the statistical analyses were performed in RStudio 4.3.1 (PBC, Boston, MA USA).

#### Calculation of eye dominance

We determined the observers’ eye dominance using the key presses during the classical binocular rivalry task in the training session. First, we computed the median percept duration for each eye and then divided the median duration of the left eye by the sum of the median percept durations of both eyes (Ding, Naber, Gayet, Van der Stigchel, & Paffen, 2018). Observers having more than 0.8 or less than 0.2 left eye dominance were immediately excluded at the end of the training session.

#### Transition types

Using the observers’ key presses during the experimental sessions, we computed the following parameters for each condition: (1) overall transition rate, defined as the number of all reported transitions regardless of the type divided by the total duration of stimulus presentation, (2) fraction of each transition type, defined as the number of occurrences of that transition type divided by the total number of reported transitions, (3) median duration of all reported transitions regardless of the type, and (4) median duration of each transition type. A change from not pressing any button to pressing one of the four buttons was counted as transition occurrence (transition onset time). Transition duration was defined as the time between pressing and releasing the same button. We removed the key presses with durations shorter than 0.1 second, as well as simultaneous key presses to reduce possible noise in the response data.

We compared the relative frequency and duration of each transition type, as well as the overall transition rate and transition duration irrespective of transition type across contrast levels. The data for high-low and low-high contrast pairs were merged. This resulted in three contrast conditions: high-high (HH), high-low (HL, regardless of which stimulus was at high contrast), low-low (LL). We performed a two-way repeated measures ANOVA using *nlme* (Pinheiro, Bates, & R Core Team, 2025; Pinheiro & Bates, 2000) and *emmeans* (Russel, 2025), modeling individual observers as random effect and the contrast condition and stimulus type as fixed effects (the latter being of no interest in this study, but reported in the Supplementary Materials for completeness). Because no interaction between stimulus type and condition was found in any of the tests across the experiments, we report the corresponding statistical results only in the Supplementary Tables. For the significant main effect of contrast, we performed paired *t*-tests with Bonferroni correction as post-hoc tests.

#### Replay analysis

We calculated the response accuracy during the replay task at the level of perceptual events (see Yilmaz et al., 2025). Specifically, for transition events, a button press for superimposed transition was considered correct; for transitions shorter than 0.1 second, we considered a button press for immediate as correct because the mixed form was perceptually invisible; for dominance, absence of a button press was considered correct. Fraction of correctly reported transitions was computed for each subject to determine potential outliers (see “Screening and exclusion criteria”).

#### Visualization

**Figure 2.**
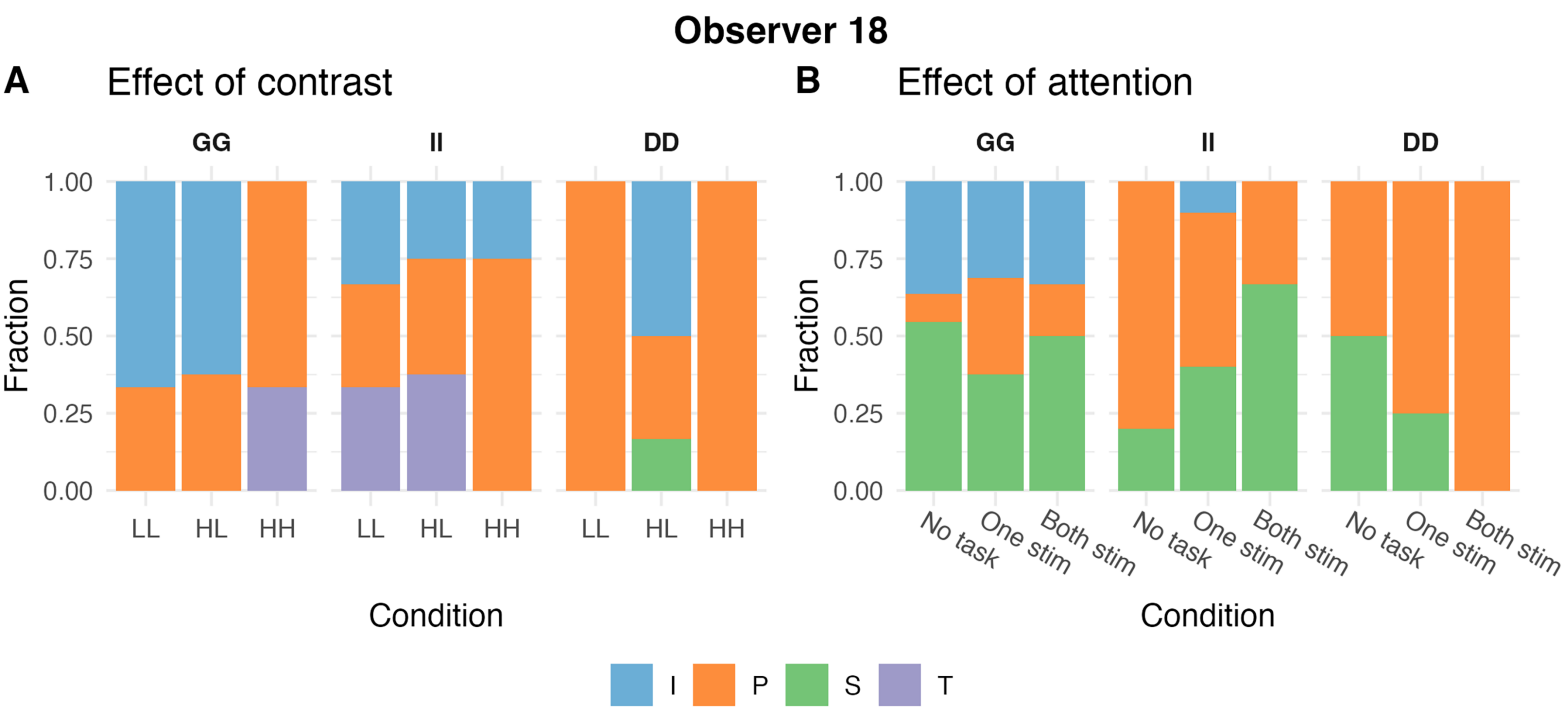
**Fraction (relative frequency) of each transition type for a sample observer.** Observer 18 experienced all four transition types across the contrast (**A**) and attention (**B**) experiments, but not within every single block. Consequently, the fractions do sum up to one for each block, but do not sum up to one when averaged over blocks with different stimuli or over observers.

**Figure 3.**
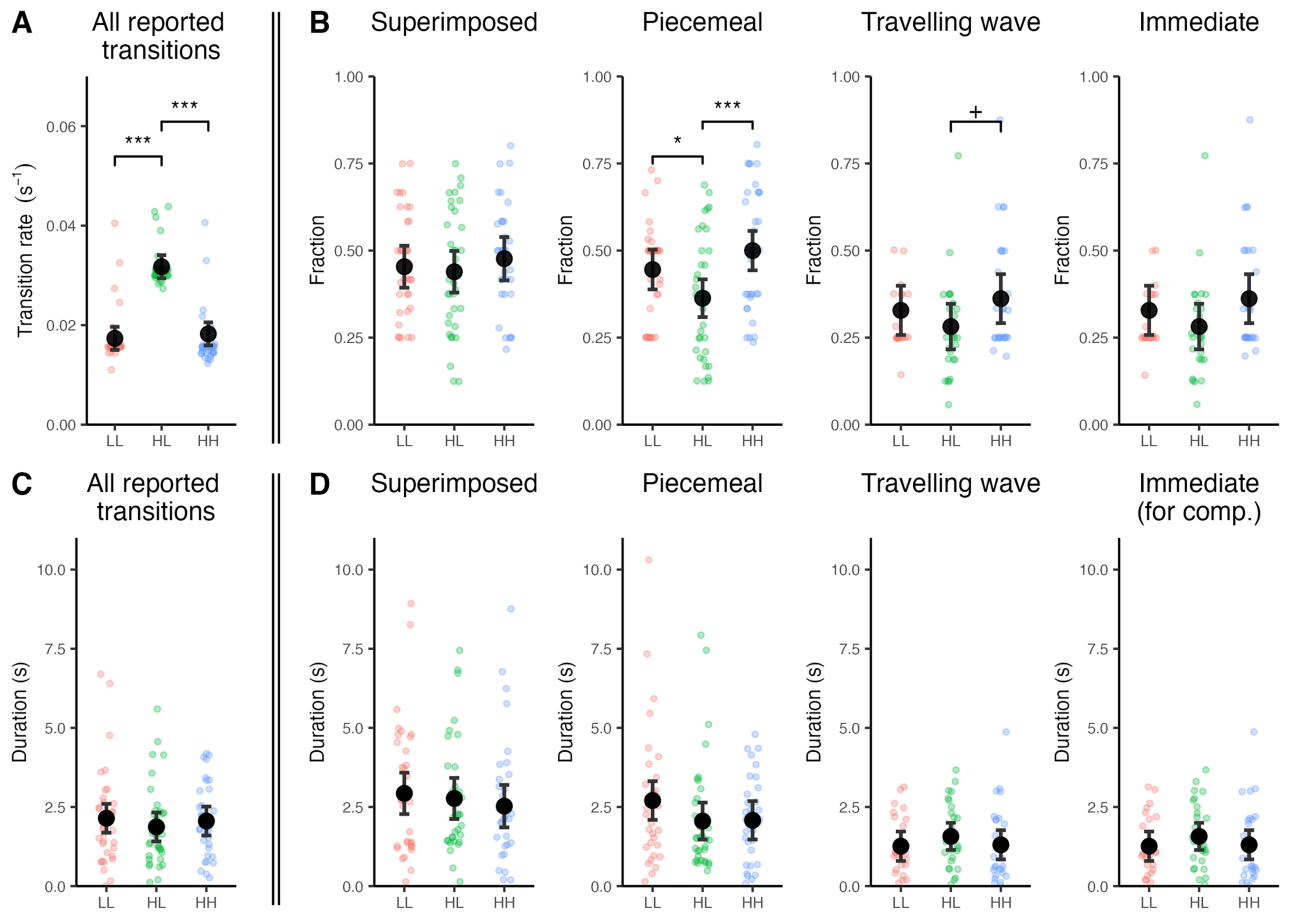
**Effect of contrast on rivalry transitions.** The plots show the frequencies and durations across contrast conditions (estimated marginal means; LL: low-low, HL: high-low, HH: high-high) with error bars indicating the 95% confidence intervals (CI), and the individual data points represents the mean value for each observer. (**A**) Overall transition rate, defined as the number of transitions per second; (**B**) Fraction (relative frequency) of each transition type; (**C**) Overall transition duration; (**D**) Duration of each transition type. The fractions of piecemeal and traveling wave transitions were significantly affected by contrast. Please note that the true duration of immediate transitions in D is 0, and what is visualized is the duration of the key press that indicated an immediate transition. *p_Bonferroni_ < 0.05, **p_Bonferroni_ < 0.01, ***p_Bonferroni_ < 0.001, +*p*_uncorrected_ < 0.05.

Because there was no significant interaction between the stimulus type and the condition, we visualized the main effect of condition in our data by using the estimated marginal means for each condition from the liner mixed model in Figure 3 and Figure 5. Therefore the error bars illustrate the 95% confidence interval of the estimated marginal means. The individual data points are added to visualize the distribution of the data, and each point represent the average value across stimulus types for each individual. The summary of the fraction and duration of each transition type per stimulus type, condition and experiment is visualized in Supplementary Figures S1–S3. Because not all transition types were perceived by each observer in every block, the fractions of transition types add up to 1 at the level of single blocks, as illustrated in Figure 2, but not when averaged over blocks of different stimulus types and not at the group level.

### Results

#### Rate and fractions of reported transitions

First, we tested the effect of contrast on the overall transition rate. The contrast level of the stimuli significantly affected the overall transition rate (*F*(2, 280) = 101.42, *p* = 0.00, Cohen's *f*^2^ = 5.8 × 10^−^^16^). As illustrated in Figure 2A, the transition rate was increased in the HL contrast condition compared to LL (*t*(280) = 12.71, *p*_Bonferroni_ = 4.6 × 10^−^^29^, Cohen's *d* = 1.52) and HH (*t*(280) = 11.92, *p*_Bonferroni_ = 2.8 × 10^−^^26^, Cohen's *d* = 1.42) with large effects. Stimulus type did not affect the transition rate (Supplementary Figure S1A), and there was no interaction between contrast level and stimulus type (see Supplementary Tables S1 and S2 for full statistical details).

Next, a key test of this study was whether contrast manipulation had an effect on the fraction of each transition type separately. Note that because we tested the effect on *fractions* of a given transition type (e.g., number of piecemeal transitions divided by all transitions), significant differences across contrasts directly imply that the given transition type changed relative to other transition types.

The contrast of the stimuli had a significant effect on the fraction of piecemeal (*F*(2, 184) = 9.88, *p* = 8.4 × 10^−^^5^, Cohen's *f*^2^ = 6.5 × 10^−^^10^) and traveling wave (*F*(2,102) = 3.50, *p* = 0.034, Cohen's *f*^2^ = 1.8 × 10^−^^12^) transitions. Piecemeal transitions were less frequent in the HL condition compared to the HH condition (*t*(184) = 4.45, *p*_Bonferroni_ = 4.4 × 10^−^^5^) with medium effect (Cohen's *d* = 0.66) and the LL condition (*t*(184) = 2.65, *p*_Bonferroni_ = 0.026) with small effect (Cohen's *d* = 0.39). Similarly, traveling wave transitions were less frequent in the HL condition compared to the HH condition (*t*(102) = 2.27, *p*_uncorrected_ = 0.025, Cohen's *d* = 0.45), but not significantly different than in the LL condition (*t*(102) = 1.29, *p*_Bonferroni_ = 0.60). There were no significant effects for other transition types and no interaction between contrast and stimulus type. These results are summarized in Figure 2B. The main effect of stimulus type, which was not of interest here, is presented in Supplementary Figure S1B (see Supplementary Tables S1 and S2 for full statistical details).

Because the contrast of stimuli had similar effect on piecemeal and traveling wave transitions, in an additional analysis we made sure that our participants indeed reported these two transition types independently. We calculated the temporal distance between the onsets of these two transition types when they were consecutively reported. Only 3.8% of all traveling wave transitions (10 data points) either followed or were followed by piecemeal transitions with an interval ranging between 2.9 and 25.8 seconds (mean and *SD* 7.5 ± 6.7 seconds). Hence, we conclude that these rare cases of traveling wave transitions after the piecemeal transitions (and vice versa) were temporally distinct events. We conclude that the effect on traveling wave transitions is not confounded by inability of subjects to distinguish them from piecemeal transitions.

#### Transition duration

The contrast level of the stimuli did not affect the overall duration of transitions (*F*(2, 280) = 0.82, *p* = 0.44). There was no significant interaction between contrast and stimulus type. Stimulus type, which was not of interest in this study, had a significant effect on the overall transition duration (*F*(2, 280) = 14.78, *p* = 7.9 × 10^−^^7^, Cohen's *f*^2^ = 1.34 × 10^−^^16^). Transition durations were shorter for grating rivalry compared to images (*t*(280) = 4.32, *p*_Bonferroni_ = 1.94 × 10^−^^4^, Cohen's *d* = −0.48) and moving dots (*t*(280) = 4.89, *p*_Bonferroni_ = 1.38 × 10^−^^6^, Cohen's *d* = 0.62) (Supplementary Figure S1C).

We further investigated the effect of contrast on the duration of each transition type separately but found no significant effects for any of the transition types. We did find a significant effect of stimulus type on the duration of superimposed (*F*(2, 212) = 4.40, *p* = 0.013, Cohen's *f*^2^ = 2.88 × 10^−^^17^) and piecemeal (*F*(2, 184) = 5.36, *p* = 0.0055, Cohen's *f*^2^ = 1.34 × 10^−16^) transitions (Supplementary Figure S1D), but none of the transition types showed an interaction between stimulus type and contrast. Full statistical results are presented in Supplementary Tables S3 and S4.

## Experiment 2: Attention

The attention experiment consisted of two task sets, both of which used identical rivalry stimuli and differed in the location of attention. In one task set, attention was directed to the checkerboard frame surrounding the rivalry stimuli (“task set on the checkerboard” with attention on or off), in the other task set, attention was directed to the rivaling stimuli (“task set on rivalry” with attention on both stimuli, one of the stimuli, or no attention). The main hypotheses, experimental procedures and data analysis strategy were preregistered before the start of the data collection on osf.io (https://osf.io/ubkjy).

### Observers

A total of 37 observers were recruited for the study. Twenty-five of them participated in both task sets, nine of them completed only the task set on the checkerboard and three of them completed only the task set on rivalry (see “Experimental design”). After applying the exclusion criteria (see below), data of 34 observers between 18 and 40 years old (*M* = 25, *SD* = 5, 26 female, eight male) were used in the final analysis of the task set on checkerboard, and data of 28 observers who were between 18 and 39 years old (*M* = 25, *SD* = 4.7, 23 female, five male) were used for the task set on rivalry. The sample size was determined with an a priori power analysis for our primary hypothesis (the effect of attention condition on transition duration and frequency) using G* Power (Faul et al., 2007; Faul et al., 2009). Because the number of conditions differed in the two task sets, power analysis was performed separately for each task set. We had three conditions for the task set on rivalry and the required sample size was 28 to reject the null hypothesis with 80% power. For the task set on the checkerboard, on the other hand, the experiment included only two conditions. Therefore we computed the required sample size with the same parameters except the number of measurements, which was two instead of three. The power analysis yielded a required sample size of 34 to be sufficient to reject the null hypothesis. All the observers gave their written informed consent and agreed with the data protection policy prior to participating. The experiment was positively assessed by the ethics committee of the University of Graz.

### Screening and exclusion criteria

As in Experiment 1, before the main experiment, observers underwent a screening procedure to make sure they fulfilled the inclusion criteria. Because of technical restrictions, the observers performed only the Freiburg Visual Acuity Test $FrACT_{10}^{2022-04-26}$ (https://michaelbach.de/ot/FrACT10/capp/index.html; Bach, 1996) as a prescreening test in Experiment 2. Three observers were excluded based on the visual acuity test with both eyes open and with the best correction, and none were excluded based on eye dominance.

### Experimental design

The experimental design was similar to Experiment 1. The observers were trained in a separate training session followed by three experimental sessions. In each experimental session, they were presented with one of the three stimulus pairs (Figure 1A) and were asked to report the four transition types by a key press, similar to the contrast experiment. Instead of contrast manipulation used in Experiment 1, we manipulated the amount and the locus of attentional resources by introducing an additional attention task either on the checkerboard frames surrounding the rivaling stimuli or on the rivalry stimuli themselves. Each session consisted of several experimental blocks, each of which was dedicated to one of the experimental conditions of the task sets described below. Each block contained two 2-minute trials separated by 15 seconds that counterbalanced stimuli across eyes.

In the task set on checkerboard, the checkerboard frames instantaneously rotated 5° clockwise, remained there for 0.5 second and rotated back (synchronously in both eyes) to attract attention of the subjects in a bottom-up fashion (Hopfinger & Mangun, 1998; Hopfinger & Mangun, 2001). There were two conditions for this task set: “no task” (i.e., transients absent throughout a block) and “task” (transients present in a block; Figure 3A). There were eight to 12 such transient events per block in task blocks. In both conditions, observers were asked to report transitions and additionally count the rotation events and report the number at the end of each block. They were not informed in advance about the possible numbers of the rotation events or if there would occur in a block. For this task set, there were eight blocks: four repetitions of each of the two conditions (4 × 2 blocks). This was followed by two replay blocks: one without task and one with task.

In the task set on rivalry, either none, one, or both of the stimuli could undergo transient changes, resulting in four conditions: “no task,” “task on stimulus A,” “task on stimulus B,” and “task on both stimuli” (Figure 3B). In parallel to the task set on checkerboard, observers were asked to report transitions and additionally to count the number of transient events that occurred within each block and to report it at the end of each block. In blocks with task, the images and gratings rotated 5° clockwise instantaneously and rotated back after 0.5 second. Transient changes occurred eight to 12 times per block. Because rotation events were not salient enough for the moving dots, the motion direction of the dot stimuli switched to the vertical motion and turned back to the original state after 0.5 seconds. In all conditions, participants were asked to report transition types. For this task set, there were eight blocks: two repetitions of each of the four conditions (2 × 4 blocks). After these eight blocks, two replay blocks were presented, one with no task and with task on both stimuli, yielding a total of 10 blocks per session.

### Data analysis

Data analysis procedure was similar to Experiment 1, but instead of different contrast conditions, we compared different conditions of each attention task set. Moreover, we computed the accuracy of reporting the attention task (number of transients per block). This was done by subtracting the actual number of transients from the reported number of transients and taking the absolute value of the result.

### Results

#### Accuracy in the attention tasks

The observers reported the number of events at the end of each block with an average absolute deviation of 0.20 ± 0.08 in the attention on checkerboard task set and 0.27 ± 0.19 in the attention on rivalry task set. The small values confirm their attentional compliance and focus during the task. The performance in the attention on checkerboard task set was significantly better than the performance in the attention on rivalry task set when attending to both rivalry stimuli (*t*(60) = 5.08, *p* = 4.0 × 10^−^^6^). Furthermore, in the attention on rivalry task set, the observers performed equally well on the attention task when attending to one and to both of the stimuli (main effect of condition: *F*(3, 297) = 1.42, *p* = 0.24).

#### Rate and relative frequency of transitions

In parallel to the contrast experiment, we first computed the rate and the duration of all transitions irrespective of their types, as well as the fraction and average duration of each of the transition types separately. Then, we compared these parameters across the attention conditions separately for each attention task set in a two-way repeated measures ANOVA, additionally modeling the effect of stimulus type.

**Figure 4.**
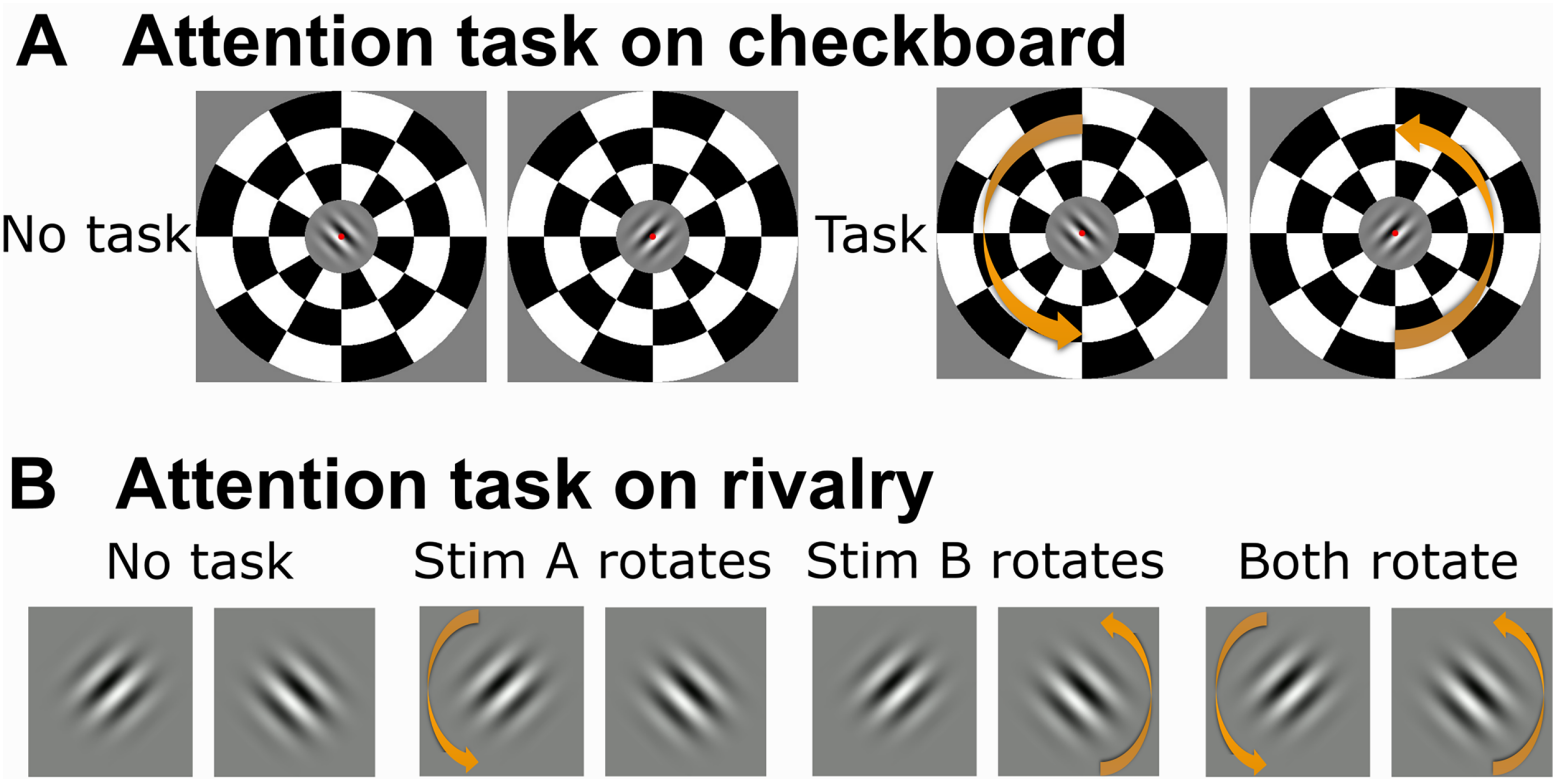
**Attention tasksets and conditions.** (**A**) In the task set on checkerboard, rivalry stimuli were presented either in the absence or in the presence of an attention task, in which the surrounding checkerboard frame patterns briefly rotated. (**B**) In the task set on rivalry, stimuli were either always static, or one or both of the stimuli rotated briefly. Observers had to count the number of events and report it at the end of each block in addition to reporting transitions (see “Experimental design” for details).

The task on the checkerboard led to no significant effect on the overall transition rate (Supplementary Table S5). On the other hand, the task on rivalry had a significant effect on the transition rate (*F*(2, 212) = 213.2, *p* = 0, Cohen's *f*^2^ = 8.04 × 10^−^^16^) without any interaction with stimulus type. Post-hoc analysis showed that the transition rate was lower for the task on one of the stimuli compared to no task (*t*(212) = 17.80, *p*_Bonferroni_ = 4.12 × 10^−^^44^, Cohen's *d* = 2.46) and compared to the task on both stimuli (*t*(212) = 17.91, *p*_Bonferroni_ = 3.13 × 10^−^^44^, Cohen's *d* = 2.45) (Figure 4^5^A), which is consistent with the corresponding contrast effects.

**Figure 5.**
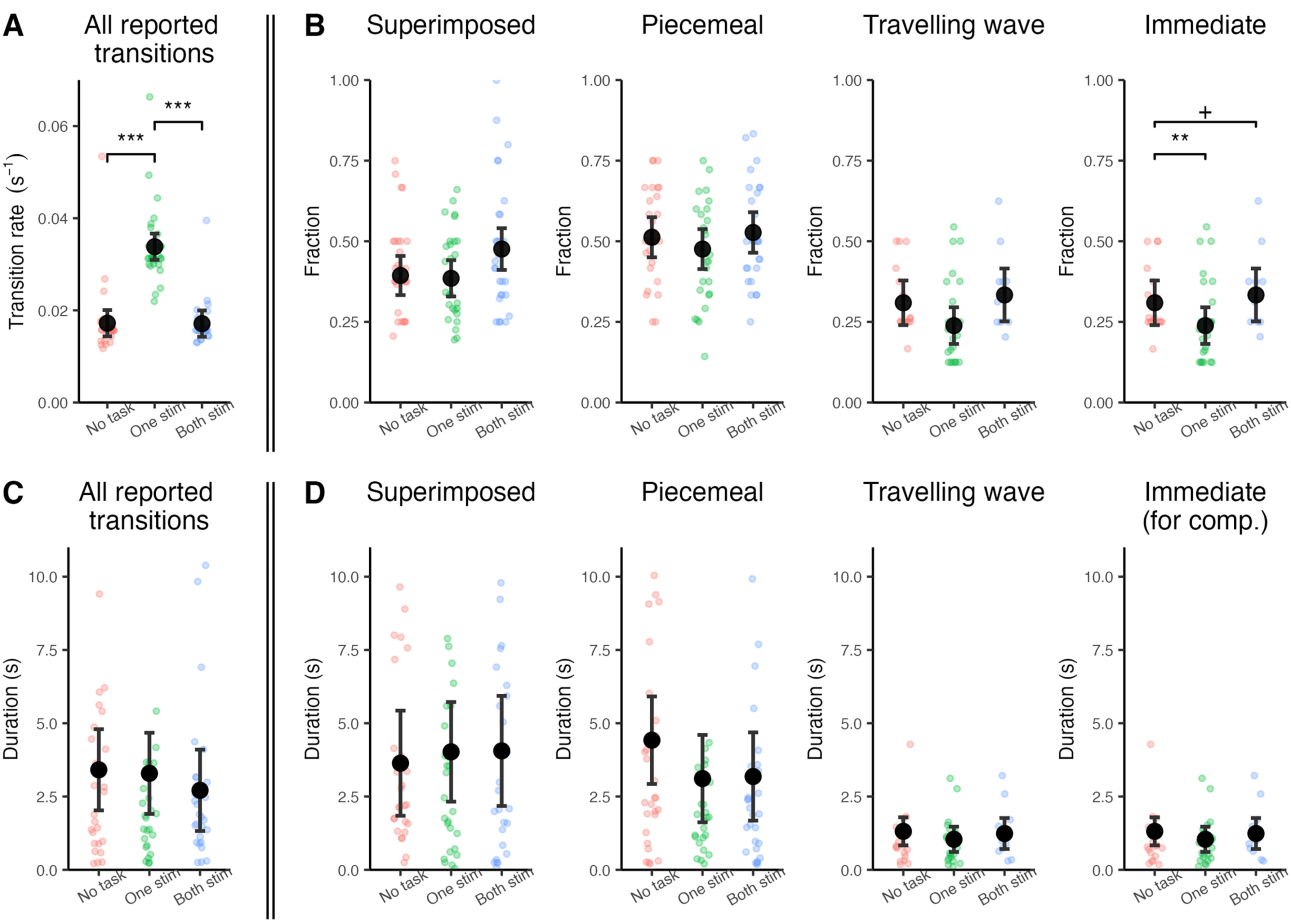
**Effect of attention on rivalry transitions.** The plots show the estimated marginal means for the frequencies and durations in different conditions of attention on rivalry task set with error bars indicating the 95% confidence intervals (CI), and the individual data points represents the mean value for each observer. (**A**) Overall transition rate, defined as the number of transitions per second; (**B**) Relative frequency of each transition type as a fraction of all transition types; (**C**) Overall transition duration; (**D**) Duration of each transition type. Please note that the true duration of immediate transitions in D is 0, and what is visualized is the duration of the key press that indicated an immediate transition. The relative frequency of immediate transitions was significantly reduced by attention. *p_Bonferroni_ < 0.05, **p_Bonferroni_ < 0.01, ***p_Bonferroni_ < 0.001, +*p*_uncorrected_ < 0.05.

We further analyzed our data to test the effect of attention tasks on the fraction of different transition types. In accord with the null effect for overall transition frequency, the task on checkerboard did not affect the relative frequency of any specific transition type (Supplementary Table S5). The task on rivalry stimuli, on the other hand, had a significant effect on the fraction of immediate transitions (*F*(2, 61) = 8.98, *p* = 3.82 × 10^−^^4^, Cohen's *f*^2^ = 1.73 × 10^−^^12^) (Figure 4B). Attention on one rivalry stimulus led to reduced fraction of immediate transitions compared to no task (*t*(61) = 3.72, *p*_Bonferroni_ = 4.32 × 10^−^^4^, Cohen's *d* = 0.95). Similarly, attention on both stimuli also showed a trend for a reduced fraction of immediate transitions compared to no task (*t*(61) = 2.05, *p*_uncorrected_ = 0.045, Cohen's *d* = 0.52). There was no interaction between condition and stimulus type. Full statistical results are presented in Supplementary Tables S5 and S7 through S9.

#### Transition duration

The overall duration of transitions was affected neither by the attentional shift away from the rivalry stimuli (*F*(1, 164) = 0.65, *p* = 0.42) nor by the attention task on rivalry stimuli (*F*(2,212) = 0.64, *p* = 0.53). The stimulus type, which is not of interest here, affected the overall duration of transitions in both task sets without any interaction with the attention condition (see the statistical summary in Supplementary Tables S6, S7, S10, and S11).

We further analyzed the effect of attention on the duration of individual transition types. The attention task sets had no effect on the duration of transition types, neither when attention was directed away from, nor toward the rivalry stimuli, which is consistent with the null effect on the overall duration of transitions. The duration of transition types was affected by the stimulus type in both attention tasks (Supplementary Figures S2 and S3). The statistical details for the main effect of stimulus type, which was not of interest here, are presented in Supplementary Tables S6, S7, S10, and S11.

## Discussion

In this study, we examined the impact of contrast and stimulus-driven attention on transition appearance in binocular rivalry. Instead of a typical binocular rivalry task, in which observers report their dominant percepts, we asked them to report the four most common transition types. While they viewed binocular rivalry and reported transitions, we manipulated either the contrast of the stimuli or the focus of attention. In agreement with prior studies (Paffen & Alais, 2011; Paffen & Van der Stigchel, 2010), we found that contrast and attention had similar effects on the overall transition rate. Importantly, our main findings additionally revealed that the relative proportions of different transition types were differentially affected by contrast and attention. These findings contradict the previously assumed similarity between attention and contrast effects in binocular rivalry (Paffen & Alais, 2011) and have important implications for neuroimaging studies of consciousness as well as for studies with neurodiverse populations. We discuss these findings and their implications in detail below.

### Contrast imbalance reduces piecemeal transitions

The first main finding of our study is that the contrast imbalance between the two stimuli primarily affected the piecemeal transitions. Piecemeal percepts were more frequent for stimuli with equal contrast and less frequent for stimuli with contrast imbalance, with a similar trend for traveling wave transitions. The similarity of the effects for piecemeal and traveling wave transitions suggests a similar perceptual mechanism underlying these two transition types. This speaks for the suggestion made in our previous study that piecemeal, traveling wave and halves transitions may represent examples of the same transition class, differing in their dynamic aspect (Yilmaz et al., 2025).

On the other hand, contrast manipulation had no effect on the duration of transition types, which is different from recent studies reporting that the duration of both superimposed and piecemeal transitions increased with the contrast level of both rivalry stimuli (Skerswetat et al., 2018a; Skerswetat et al., 2018b; Skerswetat et al., 2020). Unlike these studies, which examined only transitions between two dominant percepts, our analysis included both reversals (perceptual switch from one to the other dominant percept) and return transitions (when one percept is interrupted by a mixed percept and then returns to the original), as well as immediate changes. Therefore the results are not directly comparable.

What could be the reason for the primary effect of contrast on piecemeal transitions? Piecemeal percepts in rivalry are typically viewed as a failure of a higher-level perceptual grouping mechanism, which should act to synchronize multiple local rivalry zones (Blake et al., 1992; Kovács, Papathomas, Yang, & Fehér, 1996). The fact that contrast imbalance decreased the fraction of piecemeal transitions suggests that this higher-level perceptual grouping process may operate more effectively when one eye's input is stronger, and the visual system is in an overall less ambiguous state.

### Attention affects immediate transitions

The effect of attention on transition appearance was cardinally different from that of contrast. Surprisingly, attention manipulation primarily impacted immediate transitions. When attention was directed to either one or both stimuli, fewer immediate transitions were reported. This implies that immediate transitions are more prone to attentional influences than other transition types and may be closer linked to attentional processes in binocular rivalry. A potentially related effect was reported in a recent study, which did not distinguish between different transition types and found that stimulus-driven transient events increased the duration of transition periods (Naber, Stuit, De Kloe, Van der Stigchel, & Paffen, 2020). In our study immediate transitions could have been substituted by other transition types with nonzero duration, which would result in an overall lengthening of measured transition durations reported Naber et al. (2020).

One potential alternative explanation is that observers may have simply missed these very brief events while focusing on the attention task, as immediate transitions are most prone to inattention effects. However, this explanation appears unlikely given the overall result pattern. First, no comparable effects were observed when attention was diverted away from the rivalry stimulus. Second, within the attention on stimulus task set, inattention effects should have been stronger when observers attended to both stimuli compared to when they attended to one stimulus. However, there was no difference between the two attention conditions. Therefore the reduction in immediate transitions likely reflects true attention effects in rivalry stimuli rather than unspecific effects of missing a transition.

An alternative mechanism of the influence of attention on binocular rivalry is based on the biased competition framework (Desimone & Duncan, 1995; Dieter & Tadin, 2011). According to this view, attention should primarily affect periods of ambiguity and bias perception towards one of the representations. This idea has found empirical support in an experiment where cues primarily affected binocular rivalry dynamics at later stages of the dominance periods, when the switch was about to occur (Dieter, Melnick, whereas & Tadin, 2015). However, our results speak against the biased competition proposal. According to this proposal, attention on rivalry should reduce the duration and frequency of piecemeal transitions, which represent periods when the visual system cannot clearly decide in favor of one of the precepts, while attending away should have the opposite effect. We, however, did not observe such a pattern.

Interestingly, a complementary effect on immediate transitions was not observed when attention was diverted away from the rivalry stimulus and focused on the surrounding checkerboard frame. We also did not observe a reduction of the overall transition rate, which would be expected from the literature (Alais et al., 2010; Brascamp & Blake, 2012; Paffen et al., 2006; Zhang et al., 2011). This could indicate that diverting attention away from rivalry has either a different or a weaker effect than focusing additional attentional resources on the rivalry stimuli themselves. In the diverted attention experiments, both spatial and feature-based attentional resources are withdrawn from rivalry. In attention on rivalry experiments, on the other hand, only additional feature-based attentional resources are concentrated on the stimulus, while spatial attention is on rivalry by default (Li, Rankin, Rinzel, Carrasco, & Heeger, 2017). Influencing spatial and feature-based attention is expected to have a stronger effect than influencing feature-based attention only). The somewhat higher response accuracy on the attention task in the attend-away condition indicates that this task was either easier for our participants or that participants had put more effort into performing the task. Because we cannot distinguish the two possibilities, further dedicated studies are needed to confirm the difference between withdrawing and allocating attentional resources on the appearance of binocular rivalry transitions.

It is also possible that the stimulus transients associated with attention (but not no-attention) condition counteracted the expected attention-driven decrease of perceptual transitions rates. Prior studies have shown that stimulus transients can induce rivalry switches and hence increase transition rates (Bartels & Logothetis, 2010; Kanai, Moradi, Shimojo, & Verstraten, 2005; Wolfe, 1984). However, we did not observe an increase in the overall transition rate in attention on checkerboard task set, and also no difference between attention on both stimuli and no attention in attend-rivalry task set, making this explanation unlikely.

It is important to note that the effect of attention in our study stems primarily from the exogenous attention cued by the transient change in visual input. So far, minor differences have been reported between exogenous and endogenous attention effects on binocular rivalry: both prolong the dominance duration of attended stimulus while not affecting transition rate (Ooi & He, 1999; Paffen & Alais, 2011), although the effect of endogenous attention is weaker compared to exogenous attention (Chong & Blake, 2006). It remains unclear if the endogenous attention would have the same effect on transition appearance. It could be, analogous to the difference between contrast and attention reported in this study, that the two attention types appear similar quantitatively, but actually affect different transition types. In fact, the change in pupil size in response to endogenous attentional modulation suggests that it influences superimposed but not piecemeal transitions (Acquafredda, Binda, & Lunghi, 2022). Further studies are needed to test this hypothesis.

In any case, our results demonstrate the importance of considering non-instantaneous transitions of different types, especially when additional attentional resources are focused on binocular rivalry stimuli. The fact that under attention on rivalry more transitions are non-instantaneous is particularly relevant for neuroimaging studies of the neural correlates of consciousness, where participants are asked to focus on the stimuli and report them (the so-called explicit report paradigm). In such studies it is important to measure and simulate non-immediate transition types to achieve an accurate measure of dominance onset times and to create a perceptually and temporally comparable control replay condition.

### Similarity and difference between attention and contrast effects

Our findings demonstrate that the previously reported similarity between the effects of contrast and attention (Paffen & Alais, 2011) may not extrapolate to all aspects of rivalry phenomenology. Both contrast imbalance and attention imbalance (more attentional resources on one of the two stimuli) led to an increase in the overall rate of reported transition events. This effect was independent of the stimulus type used and may reflect an attempt of the weaker stimulus to brake the dominance phases of the stronger stimulus, which manifests itself as an increase in the fraction of return transitions (whereby the “strength” can be equally affected by contrast or by attention) (Brascamp et al., 2006). Crucially, looking at the four different transition types separately revealed important differences. Contrast manipulation affected the fraction of piecemeal and traveling wave transitions, while attention primarily affected the fraction of immediate transitions. Hence, although the effects of contrast and attention are similar quantitatively, they are qualitatively different.

Our findings of distinct effects of contrast and attention on binocular rivalry phenomenology are broadly consistent with findings in a few other careful investigations of these two effects on binocular rivalry dynamics. For example, an increase in the contrast level of the stimuli increases pupil size, while attentional modulation has no such effect on observers’ physiology (Acquafredda et al., 2022). Moreover, a newer binocular rivalry model acknowledges two types of attention effects (Li et al., 2017). In addition to enhancing the perceived stimulus contrast, which is similar to direct contrast increase in a bottom-up fashion, it includes an attentional component that regulates attention gain in a top-down fashion. In sum, other studies also revealed subtle differences between contrast and attention effects on binocular rivalry, which our results expand to a perceptual level.

### Common mechanism across stimulus types

In the current study, we presented different types of stimuli: orthogonal static gratings as the low-level stimulus, images of a house and a face as a higher-level stimulus processed along the ventral stream, and coherently moving dots as a higher-level stimulus processed along the dorsal stream. We expected to observe the effect of contrast and attention at different levels and pathways of information processing in the visual system, as it has been previously reported for flip rate and transition durations (Alais et al., 2010; Alais & Melcher, 2007). Consistent with this, we observed overall differences between stimulus types in the appearance of transitions, similar to our previous findings (Yilmaz et al., 2025). However, there were no differences in how attention and contrast affected the specific transition profile of each stimulus type. This result suggests that despite differences in representational complexity, the type of bottom-up input plays no role in how contrast and attention affect transition appearance.

### Implications for neurodevelopmental disorders

A previously reported association between the symptom severity of adult ADHD and ASD and binocular rivalry transition durations has been interpreted as the reduction of inhibition in the visual system of these individuals (Casanova et al., 2013; Jusyte et al., 2018; Robertson et al., 2013). An increased proportion of superimposed transitions in ASD group has also been reported (Skerswetat et al., 2022). In accord with this, our findings suggest that a more differentiated view of different transition types, e.g., looking specifically at the fraction of specific transition types, could be more beneficial. For example, as discussed above, the lengthening of transitions observed in ADHD and ASD could result from a reduction in the fraction of immediate transitions (Casanova et al., 2013; Dunn & Jones, 2020; Said et al., 2013; Spiegel et al., 2019). Hence, immediate transition fraction could yield a stronger association with symptom severity and act as a more precise marker of these conditions. Furthermore, our results imply that the apparent similarity between these two disorders in terms of association with transition durations does not necessarily imply a similarity in transition appearance and underlying neural mechanisms, as ADHD and ASD may involve different transition types.

### Limitations

One limitation of our study is the absence of simulated piecemeal and traveling wave transitions in the replay condition. Inclusion of all the transition types reported during the rivalry task would have been a more complete approach, especially if we aimed for a direct comparison between rivalry and replay. However, this approach would still be limited because observers presumably experience more transition types than the four types they reported in the current study (Yilmaz et al., 2025). Therefore the subjective experience of replay will always deviate from that of rivalry because of the complexity of the latter, and a perfect match between the two may be impossible. This fact should always be considered when claiming ecological validity of replay condition in binocular rivalry studies.

Furthermore, binocular rivalry reporting in our study was limited to the four transition types. Previous studies used retrospective reports (Klink et al., 2010; Yilmaz et al., 2025), key presses (Sheynin et al., 2019), or continuous joystick tracking (Skerswetat et al., 2018a; Skerswetat & Bex, 2023). However, considering the variety of perceptual states such as dominance, reversals, return transitions and false summation (Brascamp et al., 2006; Liu et al., 1992) as well as the various transition types, a comprehensive reporting paradigm which would capture this complexity remains a challenge (Wallis & Ringelhan, 2013; Yilmaz et al., 2025). The current study focused on a subset of perceptual states. Further methodological improvements in reporting binocular rivalry would allow us to capture more complex aspects of subjective visual experience of this phenomenon.

Finally, although we initially expected our experimental manipulation to affect the duration of transition phases, no such effects were found. This might be due to the overall short transition phases, especially for the case of traveling wave transitions, whose measured durations approached those of immediate transitions, which are effectively zero. Hence, at least for traveling waves, a different experimental setting is needed to reliably measure changes in their duration. This also suggests that our setup might not be optimal to accurately measure and distinguish the duration of these two transition types.

## Conclusions

Our findings demonstrate that, although there may be quantitative similarities between the effects of contrast and attention on binocular rivalry, there are qualitative differences. Although the imbalance in both contrast and attention reduces the rate of transitions, the two processes affect different transition types. This contradicts previous assumptions of the similarity between the effects of contrast and attention on binocular rivalry and highlights the differences between these two processes. Our findings thus emphasize the importance of studying the phenomenological aspects of subjective experience, which can uncover important differences that are not detectable with a simplified quantitative analysis.

## Supplementary Material

Supplement 1
