# Supplementary material for: Differential effects of attention and contrast on transition appearance during binocular rivalry
Source: J Vis. 2026 Jan 23;26(1):14. doi: 10.1167/jov.26.1.14 (PMC12854236; doi:10.1167/jov.26.1.14)
Supplement: Supplement 1 [file jovi-26-1-14_s001.pdf]

Supplementary materials for "Differential effects of attention and contrast on transition appearance during binocular rivalry"

Cemre Yilmaz[1*], Kerstin Maitz[1], Maximilian Gerschütz[1], Wilfried Grassegger[1], Anja Ischebeck[1,2], Andreas Bartels[3], Natalia Zaretskaya[1,2]

[1]Department of Psychology, University of Graz, Graz, ST, Austria

[2]BioTechMeNP-Graz, Graz, ST, Austria

[3]Department of Psychology, University of Tübingen, Tübingen, BW, Germany

[*] Corresponding author. Department of Psychology, University of Graz, Graz, ST Austria. E-mail: cemre.yilmaz@uni-graz.at

This research was funded in whole or in part by the Austrian Science Fund (FWF) 10.55776/P33322.

The authors declare no commercial interests.

Supplementary tables

Supplementary table 1. Results of repeated measures ANOVA for rate and relative frequency of transitions in the contrast experiment (Experiment 1)

	numDF	denomDF	F-value	p-value	Cohen's f^2
		Transition rate			
stimulus	2	280	2.40	.092	9.1×10^{-16}
condition	2	280	101.42	0.000	5.8×10^{-16}
stimulus × condition	4	280	1.57	.18	.011
		Fraction of superimposed			
stimulus	2	212	8.90	2.0×10^{-4}	-7.7×10^{-11}
condition	2	212	0.44	.65	-1.1×10^{-10}
stimulus × condition	4	212	1.29	.28	.016
		Fraction of piecemeal			
stimulus	2	184	3.97	.020	1.5×10^{-9}
condition	2	184	9.88	8.4×10^{-5}	6.5×10^{-10}
stimulus × condition	4	184	1.08	.37	.014
		Fraction of travelling wave			
stimulus	2	102	0.54	.58	1.9×10^{-9}
condition	2	102	3.50	.034	1.8×10^{-12}
stimulus × condition	4	102	1.42	.23	.028
		Fraction of immediate			
stimulus	2	114	10.19	8.5×10^{-5}	-4.2×10^{-16}
condition	2	114	1.13	.33	-1.7×10^{-16}
stimulus × condition	4	114	0.59	.67	.0087

Note. According to Cohen's (2013) guidelines, $0.02 \leq f^2 < 0.15$ for small effect, $0.15 \leq f^2 < 0.35$ for medium effect, $0.35 \leq f^2$ for large effect. Values below 0.02 are considered negligible (negl.).

Supplementary table 2. Results of multiple comparisons for rate and relative frequency of transitions in the contrast experiment (Experiment 1)

contrast	df	t-value	p-value	p-Bonferroni	Cohen's d
		Transition rate			
HH - HL	280	-11.92	9.4×10^{-27}	2.8×10^{-26}	-1.42
HH - LL	280	0.80	.43	1.00	.095
HL - LL	280	12.71	1.5×10^{-29}	4.6×10^{-29}	1.52
		Fraction of superimposed			
DD - GG	212	3.78	2.0×10^{-4}	6.1×10^{-4}	.52
DD - II	212	0.34	.74	1.00	.046
GG - II	212	-3.51	5.5×10^{-4}	.0016	-.48
		Fraction of piecemeal			
HH - HL	184	4.45	1.5×10^{-5}	4.4×10^{-5}	.66
HH - LL	184	1.71	.088	.26	.25
HL - LL	184	-2.65	.0086	.026	-.39
DD - GG	184	-2.78	.0060	.018	-.41
DD - II	184	-2.51	.013	.039	-.37
GG - II	184	0.37	.71	1.00	.054
		Fraction of travelling wave			
HH - HL	102	2.27	.025	.076	.45
HH - LL	102	0.88	.38	1.00	.17
HL - LL	102	-1.29	.20	.60	-.26
		Fraction of immediate			
DD - GG	114	-0.98	.33	.99	-.18
DD - II	114	3.14	.0021	.0065	.59
GG - II	114	4.28	3.9×10^{-5}	1.2×10^{-4}	.80

Note. According to Cohen's (2013) guidelines, $0.20 \le d < 0.50$ for small effect, $0.50 \le d < 0.80$ for medium effect, $0.80 \le d$ for large effect. Values below 0.20 are considered negligible (negl.).

Supplementary table 3. Results of repeated measures ANOVA for duration of all transitions and transition types in the contrast experiment (Experiment 1)

	numDF	denomDF	F-value	p-value	Cohen's f^2
		Overall duration of transitions			
stimulus	2	280	14.78	7.9×10^{-7}	-1.34×10^{-16}
condition	2	280	0.82	.44	1.19×10^{-16}
stimulus × condition	4	280	0.06	.99	-7.70×10^{-5}
		Duration of superimposed			
stimulus	2	212	4.40	.013	-2.88×10^{-17}
condition	2	212	0.79	.45	0
stimulus × condition	4	212	0.68	.61	7.5×10^{-3}
		Duration of piecemeal			
stimulus	2	184	5.36	.0055	1.34×10^{-16}
condition	2	184	2.61	.076	2.09×10^{-16}
stimulus × condition	4	184	1.65	.16	.021
		Duration of travelling wave			
stimulus	2	102	2.49	.09	1.88×10^{-9}
condition	2	102	0.78	.46	1.79×10^{-12}
stimulus × condition	4	102	1.29	.28	.027

Note. According to Cohen's (2013) guidelines, $0.02 \leq f^2 < 0.15$ for small effect, $0.15 \leq f^2 < 0.35$ for medium effect, $0.35 \leq f^2$ for large effect. Values below 0.02 are considered negligible (negl.).

Supplementary table 4. Results of multiple comparisons for duration of all transitions and transition types in the contrast experiment (Experiment 1)

contrast	df	t-value	p-value	p- Bonferroni	Cohen's d
		Overall duration of transitions			
DD - GG	280	5.16	4.61×10^{-7}	1.38×10^{-6}	.62
DD - II	280	1.11	.27	.81	.13
GG - II	280	-4.06	6.46×10^{-5}	1.94×10^{-4}	-.48
		Duration of superimposed			
DD - GG	212	2.94	.0037	.011	.40
DD - II	212	0.64	.53	1.00	.087
GG - II	212	-2.39	.018	.052	-.33
		Duration of piecemeal			
DD - GG	184	3.19	.0017	.0050	.47
DD - II	184	2.29	.023	.07	.34
GG - II	184	-1.01	.31	.94	-.15

Note. According to Cohen's (2013) guidelines, $0.20 \leq d < 0.50$ for small effect, $0.50 \leq d < 0.80$ for medium effect, $0.80 \leq d$ for large effect. Values below 0.20 are considered negligible (negl.).

Supplementary table 5. Results of repeated measures ANOVA for the task accuracy in attention tasksets.

	numDF	denomDF	F-value	p-value
		Attend-away		
task	1	165	9.2718635	2.709338e-03
stim types	2	165	0.7271622	4.848206e-01
interaction	2	165	0.4130290	6.623252e-01
		Attend-stimulus		
tasks	3	297	1.4209553	2.367200e-01
stim types	2	297	11.2627543	1.928683e-05
interaction	6	297	3.1406750	5.322625e-03
		Attention tasksets		
tasksets	1	60	25.7654133	4.002394e-06
stim types	2	120	30.6097255	1.813794e-11
interaction	2	120	59.0161932	0.000000e+00

Supplementary table 6. Results of repeated measures ANOVA for rate and relative frequency of transitions in the attention taskset on frame (Experiment 2)

	numDF	denomDF	F-value	p-value	Cohen’s f^2
			Transition rate		
stimulus	2	164	6.90	.0013	7.26×10^{-17}
condition	1	164	0.082	.78	-2.90×10^{-17}
stimulus × condition	2	164	0.17	.85	8.01×10^{-4}
			Fraction of superimposed		
stimulus	2	125	6.28	.0025	5.66×10^{-13}
condition	1	125	0.11	.74	2.40×10^{-16}
stimulus × condition	2	125	1.13	.33	.012
			Fraction of piecemeal		
stimulus	2	142	0.98	.38	-9.99×10^{-17}
condition	1	142	0.32	.57	-7.01×10^{-17}
stimulus × condition	2	142	0.24	.78	1.69×10^{-3}
			Fraction of travelling wave		
stimulus	2	56	3.00	.06	-1.21×10^{-12}
condition	1	56	0.63	.43	-7.29×10^{-13}
stimulus × condition	2	56	0.09	.91	4.52×10^{-4}
			Fraction of immediate		
stimulus	2	83	3.54	.034	1.86×10^{-12}
condition	1	83	0.002	.97	1.64×10^{-12}
stimulus × condition	2	83	0.16	.85	1.29×10^{-3}

Note. According to Cohen’s (2013) guidelines, $0.02 \le f^2 < 0.15$ for small effect, $0.15 \le f^2 < 0.35$ for medium effect, $0.35 \le f^2$ for large effect. Values below 0.02 are considered negligible (negl.).

Supplementary table 7. Results of repeated measures ANOVA for duration of all transitions and transition types in the attention taskset on frame (Experiment 2)

	numDF	denomDF	F-value	p-value	Cohen's f^2
		Overall duration of transitions			
stimulus	2	164	9.32	1.47×10^{-4}	3.14×10^{-16}
condition	1	164	0.65	.42	8.75×10^{-17}
stimulus × condition	2	164	0.54	.59	2.46×10^{-3}
		Duration of superimposed			
stimulus	2	125	9.41	1.56×10^{-4}	-3.55×10^{-18}
condition	1	125	0.10	.75	7.10×10^{-18}
stimulus × condition	2	125	0.09	.91	1.40×10^{-4}
		Duration of piecemeal			
stimulus	2	142	2.50	.09	-3.53×10^{-17}
condition	1	142	0.34	.56	-1.77×10^{-17}
stimulus × condition	2	142	0.99	.37	4.77×10^{-3}
		Duration of travelling wave			
stimulus	2	56	1.71	.19	3.46×10^{-12}
condition	1	56	0.38	.54	1.42×10^{-12}
stimulus × condition	2	56	0.26	.77	4.51×10^{-3}

Note. According to Cohen's (2013) guidelines, $0.02 \leq f^2 < 0.15$ for small effect, $0.15 \leq f^2 < 0.35$ for medium effect, $0.35 \leq f^2$ for large effect. Values below 0.02 are considered negligible (negl.).

Supplementary table 8. Results of multiple comparisons for the effect of stimulus type in the attention taskset on frame (Experiment 2)

contrast	df	t-value	p-value	p- Bonferroni	Cohen's d
		Fraction of superimposed			
DD - GG	125	3.24	.0016	.0047	.58
DD - II	125	0.21	.84	1.00	.037
GG - II	125	-3.03	.0030	.0089	-.54
		Fraction of immediate			
DD - GG	83	-0.82	.41	1.00	-.18
DD - II	83	1.75	.084	.25	.38
GG - II	83	2.67	.0092	.028	.59
		Overall duration of transitions			
DD - GG	164	4.09	6.85×10^{-5}	2.06×10^{-4}	.64
DD - II	164	0.84	.40	1.00	.13
GG - II	164	-3.26	.0013	.0040	-.51
		Duration of superimposed			
DD - GG	110	4.33	3.03×10^{-5}	9.10×10^{-5}	.77
DD - II	110	2.48	.015	.044	.44
GG - II	110	-1.99	.049	.15	.36

Note. According to Cohen's (2013) guidelines, $0.20 \leq d < 0.50$ for small effect, $0.50 \leq d < 0.80$ for medium effect, $0.80 \leq d$ for large effect. Values below 0.20 are considered negligible (negl.).

Supplementary table 9. Results of repeated measures ANOVA for rate and relative frequency of transitions in the attention taskset on rivalry stimulus (Experiment 2)

	numDF	denDF	F-value	p-value	Cohen's f^2
			Transition rate		
stimulus	2	212	3.35	.037	2.01×10^{-15}
condition	2	212	213.2	0	8.04×10^{-16}
stimulus × condition	4	212	0.34	.85	-9.18×10^{-4}
			Fraction of superimposed		
stimulus	2	150	5.12	.0065	-1.37×10^{-11}
condition	2	150	2.48	.087	-3.84×10^{-11}
stimulus × condition	4	150	1.24	.30	.021
			Fraction of piecemeal		
stimulus	2	185	1.42	.24	2.40×10^{-10}
condition	2	185	1.15	.32	2.40×10^{-10}
stimulus × condition	4	185	0.80	.53	.012
			Fraction of travelling wave		
stimulus	2	46	1.30	.28	6.03×10^{-12}
condition	2	46	3.49	.039	-3.21×10^{-11}
stimulus × condition	4	46	1.01	.41	.051
			Fraction of immediate		
stimulus	2	61	4.49	.015	2.94×10^{-13}
condition	2	61	8.98	3.82×10^{-4}	-1.73×10^{-12}
stimulus × condition	4	61	0.52	.72	.011

Note. According to Cohen's (2013) guidelines, $0.02 \leq f^2 < 0.15$ for small effect, $0.15 \leq f^2 < 0.35$ for medium effect, $0.35 \leq f^2$ for large effect. Values below 0.02 are considered negligible (negl.).

Supplementary table 10. Results of multiple comparisons for rate and relative frequency of transitions in the attention taskset on rivalry stimulus (Experiment 2)

contrast	df	t-value	p-value	p- Bonferroni	Cohen's d
		Transition rate			
Both - No task	212	-0.099	.92	1.00	-.014
Both - One stim	212	-17.89	3.13×10^{-44}	9.38×10^{-44}	-2.46
No task - One stim	212	-17.80	4.12×10^{-44}	1.24×10^{-43}	-2.45
		Fraction of superimposed			
DD - GG	150	2.57	.011	.033	.42
DD - II	150	0.72	.47	1.00	.12
GG - II	150	-1.99	.04	.15	-.32
		Fraction of immediate			
Both - No task	61	-2.05	.045	.14	-.52
Both - One stim	61	1.64	.11	.32	.42
No task - One stim	61	3.72	4.32×10^{-4}	.0013	.95
DD - GG	61	1.11	.27	.81	.28
DD - II	61	2.60	.012	.035	.67
GG - II	61	1.83	.073	.22	.47

Note. According to Cohen's (2013) guidelines, $0.20 \leq d < 0.50$ for small effect, $0.50 \leq d < 0.80$ for medium effect, $0.80 \leq d$ for large effect. Values below 0.20 are considered negligible (negl.).

Supplementary table 11. Results of repeated measures ANOVA for duration of all transitions and transition types in the attention taskset on rivalry stimulus (Experiment 2)

	numDF	denDF	F-value	p-value	Cohen's f^2
Overall duration of transitions					
stimulus	2	212	13.70	2.54×10^{-6}	-9.15×10^{-17}
condition	2	212	0.64	.53	3.05×10^{-17}
stimulus × condition	4	212	1.25	.29	.014
Duration of superimposed					
stimulus	2	150	13.15	5.46×10^{-6}	-9.21×10^{-7}
condition	2	150	0.14	.87	-9.21×10^{-7}
stimulus × condition	4	150	0.56	.69	7.17×10^{-3}
Duration of piecemeal					
stimulus	2	185	9.09	1.71×10^{-4}	-4.53×10^{-17}
condition	2	185	2.15	.12	-9.06×10^{-17}
stimulus × condition	4	185	1.30	.27	.016
Duration of travelling wave					
stimulus	2	46	0.74	.48	-2.15×10^{-17}
condition	2	46	0.98	.38	0
stimulus × condition	4	46	0.33	.86	7.25×10^{-3}

Note. According to Cohen's (2013) guidelines, $0.02 \leq f^2 < 0.15$ for small effect, $0.15 \leq f^2 < 0.35$ for medium effect, $0.35 \leq f^2$ for large effect. Values below 0.02 are considered negligible (negl.).

Supplementary table 12. Results of multiple comparisons for duration of all transitions and transition types in the attention taskset on rivalry stimulus (Experiment 2)

contrast	df	t-value	p-value	p- Bonferroni	Cohen's d
		Overall duration of transitions			
DD – GG	212	5.02	1.08×10^{-6}	3.25×10^{-6}	.69
DD – II	212	1.30	.20	.59	.18
GG – II	212	-3.75	2.27×10^{-4}	6.80×10^{-4}	-.51
		Duration of superimposed			
DD – GG	150	4.62	8.12×10^{-6}	2.44×10^{-5}	.75
DD – II	150	3.54	5.98×10^{-4}	1.61×10^{-3}	.58
GG – II	150	-1.65	.10	.30	-.27
		Duration of piecemeal			
DD - GG	185	3.54	5.01×10^{-4}	.0015	.52
DD - II	185	-0.01	.99	1.00	-.0015
GG - II	185	-3.72	2.67×10^{-4}	8.04×10^{-4}	-.55

Note. According to Cohen's (2013) guidelines, $0.20 \leq d < 0.50$ for small effect, $0.50 \leq d < 0.80$ for medium effect, $0.80 \leq d$ for large effect. Values below 0.20 are considered negligible (negl.).

Supplementary table 13. Summary of the data from the contrast experiment.

Percept pair	Number of observers			Duration of first percept (s)			Relative frequency		
	GG	II	DD	GG	II	DD	GG	II	DD
				Low-Low					
S-NP	22	28	30	2.39±1.64	3.43±3.10	3.50±2.82	0.34±0.15	0.51±0.22	0.47±0.22
P-NP	24	21	21	2.12±1.78	3.02±2.68	4.06±4.26	0.44±0.21	0.43±0.16	0.41±0.21
T-NP	15	8	11	1.21±1.11	1.28±1.53	2.16±2.30	0.30±0.12	0.36±0.17	0.31±0.14
I-NP	23	9	18	0.62±1.64	0.97±1.34	0.81±1.55	0.37±0.21	0.31±0.17	0.30±0.11
NP-S	23	30	29	2.88±2.35	3.73±2.88	5.37±4.32	0.36±0.17	0.56±0.26	0.48±0.23
NP-P	24	22	22	2.93±1.34	4.60±3.58	5.03±5.78	0.48±0.20	0.49±0.20	0.40±0.22
NP-T	14	11	12	2.81±1.91	2.81±1.79	4.05±4.62	0.36±0.22	0.36±0.16	0.35±0.23
NP-I	24	9	17	2.76±1.92	5.71±6.97	5.45±5.25	0.38±0.22	0.31±0.17	0.38±0.22
				High-Low					
S-NP	25	30	33	2.55±3.03	2.91±3.12	2.89±1.73	0.34±0.19	0.41±0.24	0.47±0.22
P-NP	27	29	20	1.46±0.90	2.28±2.39	2.79±2.43	0.33±0.21	0.34±0.19	0.32±0.16
T-NP	17	14	18	1.41±0.90	1.89±1.35	1.92±1.51	0.28±0.19	0.32±0.19	0.26±0.19
I-NP	29	17	15	0.53±0.87	0.91±1.54	2.89±7.53	0.35±0.22	0.21±0.12	0.33±0.20
NP-S	24	31	33	3.62±2.50	2.91±1.66	5.28±4.18	0.37±0.20	0.45±0.26	0.47±0.23
NP-P	28	29	22	2.99±2.40	4.03±2.35	5.09±3.27	0.34±0.18	0.41±0.23	0.29±0.15
NP-T	18	16	18	2.62±1.63	4.12±3.49	5.43±4.96	0.28±0.18	0.32±0.22	0.27±0.17
NP-I	29	17	17	2.93±1.79	3.75±3.02	6.68±6.53	0.36±0.22	0.24±0.14	0.27±0.20
				High-High					
S-NP	21	25	27	1.53±1.39	3.10±3.31	3.28±2.21	0.41±0.22	0.44±0.22	0.50±0.26
P-NP	20	26	23	2.03±1.69	1.99±1.66	2.52±2.06	0.50±0.23	0.48±0.18	0.38±0.22
T-NP	16	7	11	1.25±1.00	0.73±0.90	2.39±2.39	0.42±0.21	0.31±0.17	0.34±0.15
I-S	0	0	1	---	---	0.14	---	---	0.33
I-NP	15	9	7	0.56±0.78	0.63±1.06	1.93±2.31	0.38±0.16	0.26±0.12	0.43±0.19
NP-S	21	29	27	2.00±1.25	3.75±3.27	3.41±2.76	0.41±0.21	0.50±0.26	0.53±0.24
NP-P	20	29	22	2.47±1.89	2.21±1.39	3.23±3.05	0.60±0.24	0.50±0.18	0.47±0.26
NP-T	19	8	13	2.48±1.66	2.13±1.44	2.89±2.53	0.44±0.24	0.34±0.27	0.40±0.23
NP-I	15	11	9	2.61±2.06	4.01±2.48	2.56±1.79	0.38±0.16	0.28±0.11	0.43±0.21

Note. S: Superimposed, P: Piecemeal, T: Travelling wave, I: Immediate, NP: No Press. Each row shows values of each reported perceptual state under the consideration of the following state. E.g., row “S-NP” shows data for superimposed transitions that were followed by no button press.

Supplementary table 14. Summary of the data from the attention taskset on frame experiment.

Percept pair	Number of observers			Duration of first percept (s)			Relative frequency		
	GG	II	DD	GG	II	DD	GG	II	DD
No task									
S-I	1	0	0	0.22	---	---	0.09	---	---
S-NP	23	27	28	1.35±1.16	3.28±4.94	5.36±6.89	0.25±0.14	0.29±0.20	0.37±0.23
P-S	0	1	0	---	0.13	---	---	0.11	---
P-NP	29	28	26	2.06±1.96	3.62±4.52	3.57±2.79	0.43±0.22	0.46±0.26	0.41±0.27
T-NP	21	12	9	1.11±0.69	1.42±1.11	1.31±1.09	0.25±0.18	0.18±0.09	0.19±0.08
I-S	1	0	0	0.18	---	---	0.09	---	---
I-P	0	0	1	---	---	0.03	---	---	0.13
I-NP	24	17	21	0.40±0.67	0.42±0.60	0.49±0.50	0.26±0.18	0.24±0.14	0.22±0.15
NP-S	24	28	29	2.39±1.65	3.80±5.07	4.37±4.40	0.26±0.16	0.33±0.23	0.40±0.26
NP-P	29	31	29	2.73±3.10	2.77±1.79	3.04±2.03	0.47±0.21	0.48±0.27	0.42±0.27
NP-T	23	14	9	2.45±1.55	2.41±1.59	4.05±2.83	0.26±0.17	0.20±0.10	0.19±0.09
NP-I	24	19	22	2.20±1.44	4.14±5.79	4.14±4.01	0.27±0.17	0.22±0.13	0.25±0.17
Task									
S-NP	22	25	26	1.30±0.91	3.33±4.74	7.31±11.88	0.27±0.13	0.36±0.17	0.35±0.22
P-NP	29	29	29	2.39±2.64	2.76±2.78	3.54±6.03	0.43±0.25	0.45±0.21	0.44±0.25
T-P	0	0	1	---	---	0.34	---	---	0.11
T-NP	16	13	11	1.29±0.84	1.79±1.19	1.11±0.98	0.27±0.18	0.20±0.14	0.20±0.12
I-NP	21	14	16	0.43±0.74	0.45±0.79	0.41±0.38	0.25±0.15	0.22±0.09	0.25±0.15
NP-S	23	26	27	3.12±1.50	2.01±1.21	3.87±2.27	0.28±0.13	0.43±0.21	0.37±0.21
NP-P	31	30	30	2.79±1.84	2.80±2.09	3.65±3.38	0.45±0.23	0.50±0.22	0.46±0.24
NP-T	17	14	13	2.51±1.66	3.19±3.64	5.81±7.05	0.29±0.20	0.22±0.17	0.18±0.11
NP-I	22	16	16	2.44±1.46	3.65±2.47	3.11±2.12	0.29±0.18	0.21±0.11	0.29±0.19

Note. S: Superimposed, P: Piecemeal, T: Travelling wave, I: Immediate, NP: No Press. Convention and abbreviations as in supplementary table 13.

Supplementary table 15. Summary of the data from the attention taskset on rivalry experiment

Percept pair	Number of observers			Duration of first percept (s)			Relative frequency		
	GG	II	DD	GG	II	DD	GG	II	DD
No task									
S-I	1	0	0	0.11	---	---	0.17	---	---
S-NP	11	22	16	1.77±1.53	3.44±4.28	6.17±6.41	0.30±0.14	0.41±0.20	0.40±0.18
P-T	0	0	1	---	---	0.41	---	---	0.08
P-NP	23	24	21	2.00±1.33	6.93±8.49	5.57±5.69	0.41±0.17	0.52±0.20	0.48±0.21
T-NP	12	3	5	1.12±1.11	0.78±1.04	1.48±2.20	0.31±0.10	0.32±0.16	0.35±0.15
I-NP	10	3	9	0.30±0.23	0.79±0.72	0.49±0.52	0.48±0.29	0.33±0.14	0.63±0.37
NP-S	14	23	18	2.22±1.92	4.77±5.88	4.97±8.03	0.32±0.16	0.42±0.21	0.40±0.16
NP-P	24	25	22	2.50±1.90	4.11±4.10	4.18±5.51	0.49±0.22	0.58±0.24	0.49±0.20
NP-T	15	5	6	2.36±2.16	1.84±1.44	2.24±0.96	0.35±0.11	0.27±0.14	0.36±0.17
NP-I	11	3	10	3.81±4.89	5.05±4.21	9.02±15.96	0.43±0.28	0.25±0	0.59±0.37
One stimulus									
S-NP	17	24	23	1.56±1.74	4.53±4.28	8.44±11.20	0.22±0.14	0.35±0.22	0.41±0.22
P-NP	26	25	21	2.54±3.70	3.99±4.73	4.50±6.66	0.44±0.19	0.42±0.24	0.44±0.20
T-NP	18	7	5	1.02±0.84	2.35±2.14	1.13±0.91	0.22±0.12	0.20±0.07	0.22±0.11
I-NP	18	8	11	0.32±0.25	1.15±1.43	0.84±0.88	0.25±0.18	0.16±0.08	0.30±0.17
NP-S	18	26	24	2.09±1.65	2.49±2.00	4.45±3.91	0.26±0.14	0.43±0.24	0.43±0.21
NP-P	27	26	21	2.71±1.86	1.65±0.97	3.05±2.49	0.47±0.16	0.46±0.25	0.48±0.20
NP-T	17	8	7	3.23±2.28	2.57±2.88	10.08±16.95	0.27±0.14	0.23±0.12	0.21±0.10
NP-I	18	9	11	3.38±3.79	7.74±11.22	4.99±5.79	0.29±0.18	0.18±0.10	0.31±0.21
Both stimulus									
S-NP	10	20	19	1.26±0.95	3.93±4.63	6.30±5.76	0.43±0.20	0.48±0.26	0.48±0.25
P-S	0	0	1	---	---	0.50	---	---	0.17
P-NP	25	20	19	1.82±2.11	5.02±6.66	3.91±3.84	0.43±0.20	0.46±0.24	0.45±0.19
T-NP	8	4	2	1.16±1.22	1.77±1.01	1.14±0.81	0.43±0.21	0.47±0.38	0.29±0.06
I-NP	14	4	9	0.39±0.59	0.22±0.06	0.93±1.01	0.32±0.11	0.31±0.13	0.40±0.15
NP-S	11	23	20	1.81±0.96	2.91±3.24	6.23±10.05	0.30±0.11	0.47±0.25	0.47±0.22
NP-P	27	21	22	2.52±1.77	2.41±1.92	5.20±5.04	0.42±0.16	0.54±0.21	0.51±0.27
NP-T	8	5	2	2.70±2.66	2.95±2.37	1.77±0.66	0.48±0.23	0.43±0.35	0.29±0.06
NP-I	17	5	10	1.79±1.47	3.44±4.81	10.49±15.96	0.35±0.13	0.28±0.13	0.32±0.11

Note. S: Superimposed, P: Piecemeal, T: Travelling wave, I: Immediate, NP: No Press. Convention and abbreviations as in supplementary table 13.

Supplementary table 16. Proportion of total duration of other percepts when observers did not press any button to the reported transition types, averaged across observers and reported with standard error of mean (SEM).

Condition	Proportion of other percepts (no press)
Contrast experiment	
LL	77.8±0.2%
HL	79.0±0.2%
HH	75.6±0.2%
Attention taskset on frame	
No task	77.5±0.2%
Task	77.3±0.2%
Attention taskset on rivalry	
No task	76.0±0.4%
One stimulus	75.2±0.4%
Both stimuli	74.9±0.3%

Supplementary figures

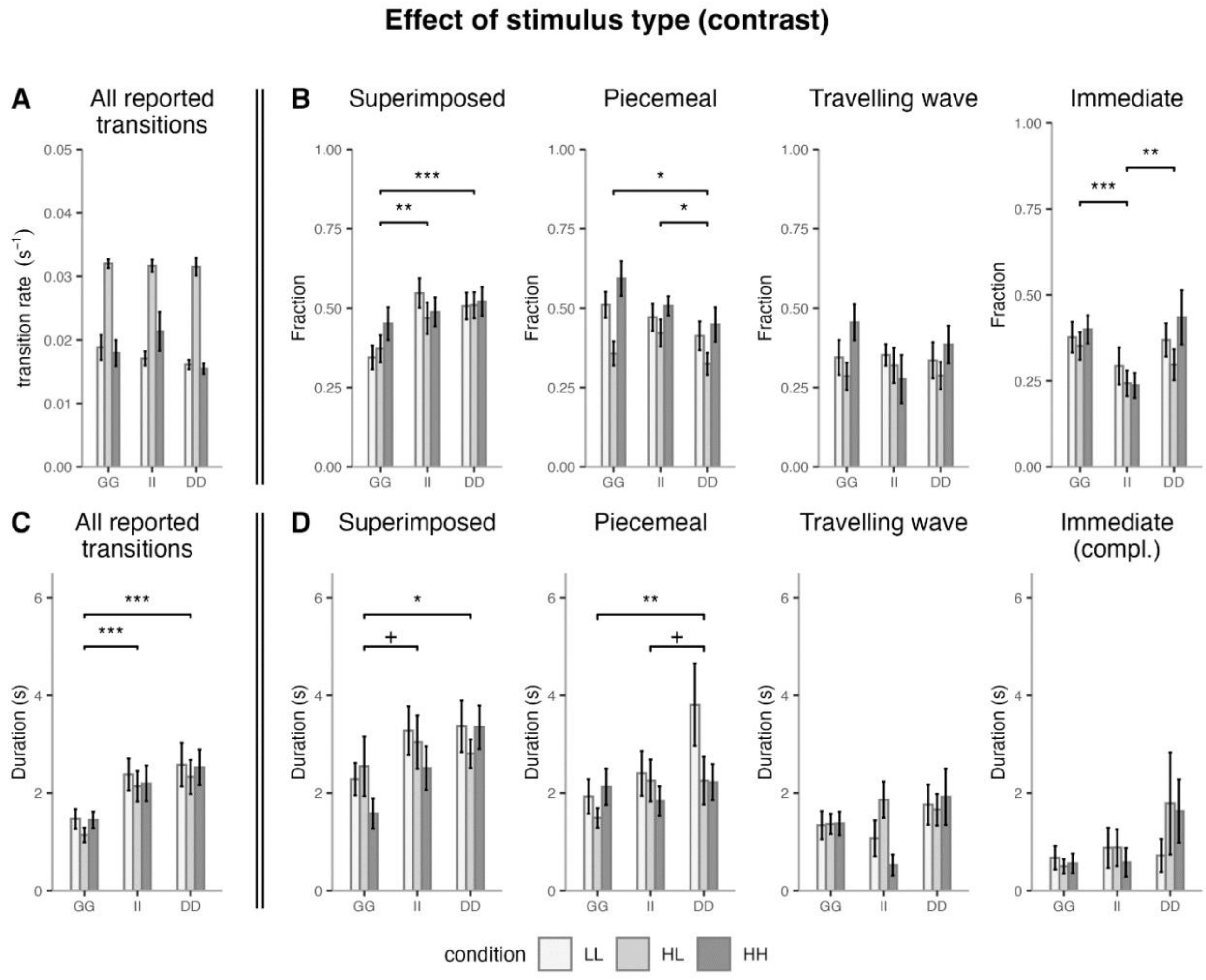

Supplementary figure 2. Effect of stimulus type across contrast conditions. The bar plots show the effect of stimulus type on the average frequencies and durations across contrast conditions with error bars indicating the standard error of the mean (SEM). A) Overall transition rate, defined as the number of transitions per second; B) Relative frequency of each transition type; C) Duration of the no-press percepts and overall transition duration; D) Duration of each transition type. The relative frequencies of superimposed, piecemeal and immediate transitions as well as the duration of superimposed and piecemeal transitions were significantly affected by stimulus type. $*p_{Bonferroni} < .05$, $**p_{Bonferroni} < .01$, $***p_{Bonferroni} < .001$, $+p_{uncorrected} < .05$.

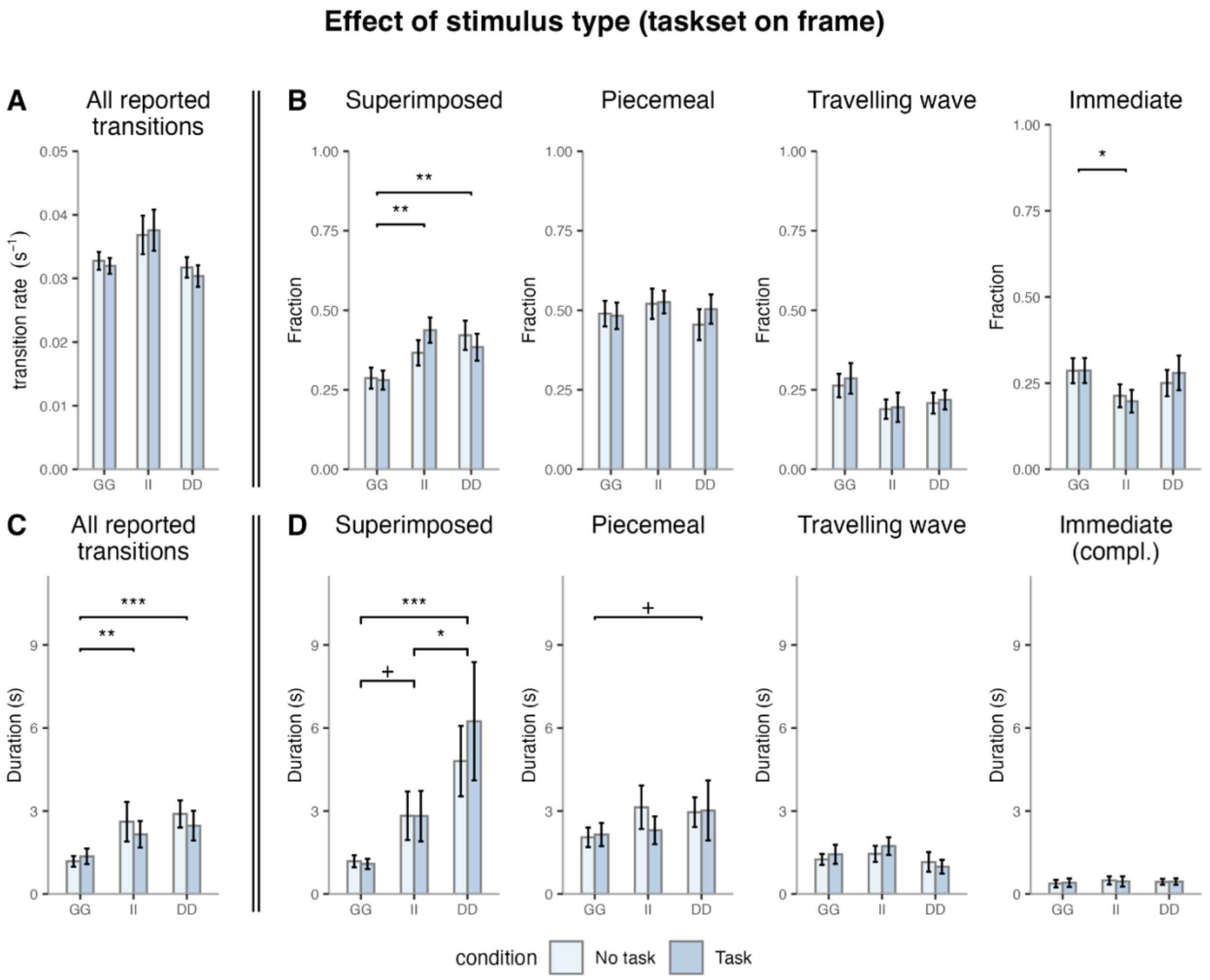

Supplementary figure 4. Effect of stimulus type across conditions of attention on surrounding fusion frame. The bar plots show the effect of stimulus type on the average frequencies and durations across conditions of the attention taskset on frame with error bars indicating the standard error of the mean (SEM). A) Overall transition rate, defined as the number of transitions per second; B) Relative frequency of each transition type; C) Duration of the no-press percepts and overall transition duration; D) Duration of each transition type. The relative frequency of superimposed and immediate transitions as well as the duration of superimposed transitions were significantly affected by stimulus type. *$p_{Bonferroni} < .05$, **$p_{Bonferroni} < .01$, ***$p_{Bonferroni} < .001$, +$p_{uncorrected} < .05$.

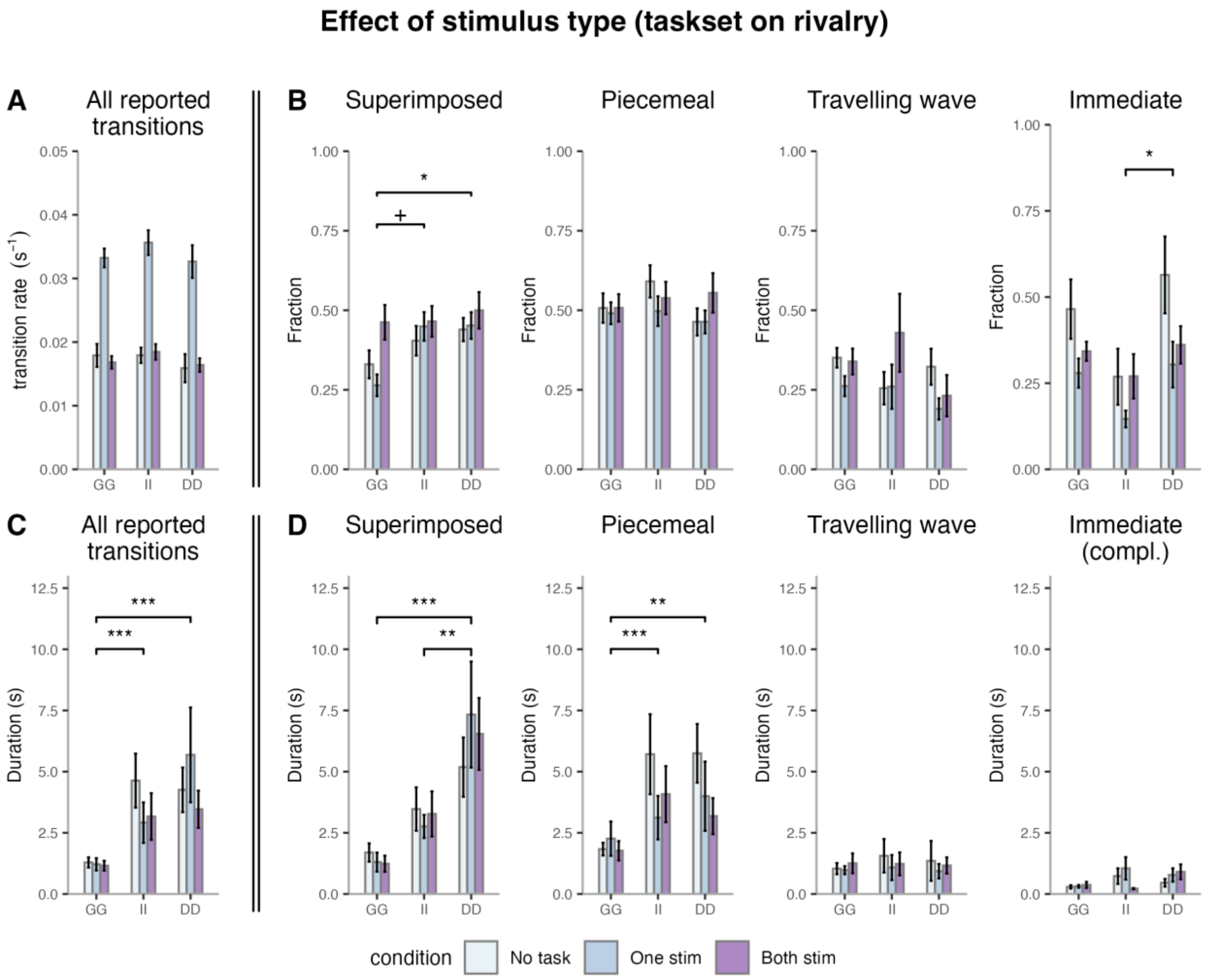

Supplementary figure 6. Effect of stimulus type across conditions of attention on rivalry stimuli. The bar plots show the effect of stimulus type on the average frequencies and durations across conditions of the attention taskset on rivalry with error bars indicating the standard error of the mean (SEM). A) Overall transition rate, defined as the number of transitions per second; B) Relative frequency of each transition type; C) Duration of the no-press percepts and overall transition duration; D) Duration of each transition type. The relative frequency of superimposed and immediate transition as well as the duration of superimposed and piecemeal transitions were significantly affected by stimulus type. *$p_{Bonferroni} < .05$, **$p_{Bonferroni} < .01$, ***$p_{Bonferroni} < .001$, +$p_{uncorrected} < .05$.

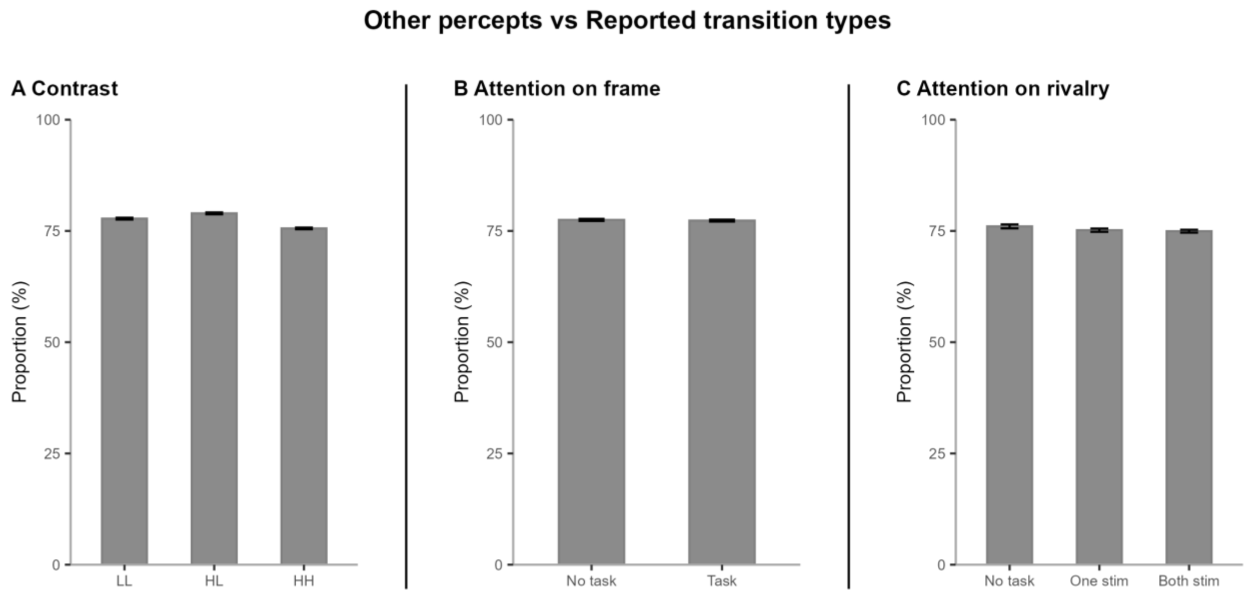

Supplementary figure 4. Proportion of time spent experiencing other percepts (absence of key press) relative to the total stimulus viewing time. The bar plots show the average proportion of the time without a key press during the experiment across observers with error bars indicating the standard error of the mean (SEM). A) Experiment 1 with different contrast conditions (LL: low-low, HL: high-low, and HH: high-high contrast pairs); B) Experiment 2 with attention taskset on frame (No task and Task conditions); C) Experiment 2 with attention taskset on rivalry stimuli (No task, Task on one stimulus, and Task on both stimuli).
